# Morphological and metabolomics profiling of intraspecific *Arabidopsis* hybrids in relation to biomass heterosis

**DOI:** 10.1038/s41598-023-36618-y

**Published:** 2023-06-12

**Authors:** Quynh Thi Ngoc Le, Naoya Sugi, Masaaki Yamaguchi, Touko Hirayama, Makoto Kobayashi, Yutaka Suzuki, Miyako Kusano, Hiroshi Shiba

**Affiliations:** 1 Graduate School of Life and Environmental Sciences, University of Tsukuba, 1-1-1 Ten-Nodai, Tsukuba, Ibaraki Japan; 2 Thuyloi University, 175 Tay Son, Dong Da, Hanoi, Viet Nam; 3 Kihara Institute for Biological Research, Yokohama City University, Yokohama, Kanagawa Japan; 4 Degree Programs in Life and Earth Sciences, Graduate School of Science and Technology, University of Tsukuba, 1-1-1 Ten-Nodai, Tsukuba, Ibaraki Japan; 5 RIKEN Center for Sustainable Resource Science, Suehiro 1-7-22, Tsurumi, Yokohama, Japan; 6 Department of Computational Biology and Medical Sciences, Graduate School of Frontier Sciences, The University of Tokyo, 5-1-5 Kashiwanoha, Kashiwa, Japan; 7 Tsukuba-Plant Innovation Research Center, University of Tsukuba, Ten-Nodai 1-1-1, Tsukuba, Ibaraki Japan

**Keywords:** Biochemistry, Molecular biology, Plant sciences

## Abstract

Heterosis contributes greatly to the worldwide agricultural yield. However, the molecular mechanism underlying heterosis remains unclear. This study took advantage of *Arabidopsis* intraspecific hybrids to identify heterosis-related metabolites. Forty-six intraspecific hybrids were used to examine parental effects on seed area and germination time. The degree of heterosis was evaluated based on biomass: combinations showing high heterosis of F_1_ hybrids exhibited a biomass increase from 6.1 to 44% over the better parent value (BPV), whereas that of the low- and no-heterosis hybrids ranged from − 19.8 to 9.8% over the BPV. Metabolomics analyses of F_1_ hybrids with high heterosis and those with low one suggested that changes in TCA cycle intermediates are key factors that control growth. Notably, higher fumarate/malate ratios were observed in the high heterosis F_1_ hybrids, suggesting they provide metabolic support associated with the increased biomass. These hybrids may produce more energy-intensive biomass by speeding up the efficiency of TCA fluxes. However, the expression levels of TCA-process-related genes in F_1_ hybrids were not associated with the intensity of heterosis, suggesting that the post-transcriptional or post-translational regulation of these genes may affect the productivity of the intermediates in the TCA cycle.

## Introduction

The growth of the world population has heightened the need to improve agricultural productivity to ensure food security. Hybrids with optimal combinations have superior performance regarding biomass, yield, or development rate and can significantly contribute to solving this problem^1^. Heterosis (or hybrid vigor) is an important phenomenon in agriculture that has been researched in various crops, including rice, wheat, maize, sorghum, sugar beet, cotton, and canola^2–6^.

Although many studies have used natural variation in intraspecific hybrids to understand heterotic mechanisms^7–11^, the correlation between genetic distance and heterosis remains a subject of debate. In maize, crosses with a greater genetic distance between parents have improved heterosis^12^. Conversely, in *Arabidopsis*, the lack of a relationship between genetic distance and the heterosis level of the parents concerning the dry weight of the shoots^13^ or the fresh weight recorded during the vegetative growth phase has been reported^7^. A variation in heterosis levels in terms of biomass was detected within reciprocal hybrids, even if the parental accessions were closely related, as in the case of the Columbia (Col), C24, Landsberg *erecta* (L*er*), and Wassilewskija (Ws)^7,9^ *Arabidopsis* ecotypes.

The analytical techniques used in metabolomics, including gas chromatography time-of-flight mass spectrometry (GC-TOF-MS), have resulted in a relatively broad coverage of compound classes that can be rapidly detected in tissues. This provides opportunities for profiling different metabolite levels between hybrids and their parents, and it may afford useful information regarding heterosis in plants^4,14,15^. A diallel cross population in rice was generated by crossing 18 inbred lines, with the metabolic profiles of the parents being used to determine three predicted agronomic traits, namely, heading date, grain yield, and plant height, using the parental metabolic profiles^4^. In *Arabidopsis*, a recombinant inbred line population generated from a cross between Col and C24 exhibited transgressive segregating biomass. The use of such a population in a metabolomics approach was based on the assumption of a high correlation between the growth rate and metabolic profiles^14^.

Several known metabolites in the central metabolic pathway are negatively correlated with biomass. These include sucrose-, glucose-, and fructose-6-phosphate; the intermediates of the tricarboxylic acid (TCA) cycle (such as citrate, succinate, malate, and 2-oxoglutarate (2-OG)); and amino acids (such as glutamine and phenylalanine)^14,16^. The TCA cycle has emerged as a central mitochondrial hub that drives the production of the ATP used in photosynthesis optimization^17^. Riewe et al. and Le et al.^18,19^ reported an association between increased fumarate/malate ratios and advantageous growth development . However, the relationship between hybrid vigor levels and changes in central metabolites in multiple intraspecific hybrids of *Arabidopsis* remains unknown.

We investigated the physiological characteristics of seed area, germination time, and vegetative growth phenotypes in multiple intraspecific lines of *Arabidopsis thaliana* at matched germination times. The establishment of a new cultivation system was an attempt to detect differences in heterosis levels in parental combinations with minimal effects on the seed size and germination time on growth at the seedling stage. Four intraspecific combinations, i.e., Col × C24, Col × Rld-1, Col × Ws-0, and Col × L*er*-1, with different levels of hybrid vigor were selected for comparative metabolomics analysis between the intraspecific hybrids and their parental lines to identify changes in metabolites associated with heterosis. Gene expression variations related to changes in metabolites were also investigated. This study is important for elucidating the relationship between TCA intermediates and heterosis levels regarding biomass and clarifying the metabolic information of intraspecific *Arabidopsis* hybrids.

## Results

### Physiological characteristics of intraspecific *Arabidopsis* hybrids

#### Selection of intraspecific hybrid combinations that were expected to have varying heterosis levels

Heterosis can be calculated as the percentage increase in the trait value of the F_1_ hybrid over the average trait value of the two parents (mid-parent value, MPV) or the trait value of the better-performance parent (better-parent value, BPV). As a result, it is frequently ideal to compare a hybrid's performance to both BPV and MPV. For a hybrid to be considered commercially advantageous, it typically needs to outperform the BPV for agronomically significant characteristics like vegetative biomass or seed yield.

The 202 intraspecific lines (Dataset S1) used in this experiment were self-fertilized via hydroponics under uniform conditions to reduce interindividual variation during germination and growth^19^. Differences in flowering time among intraspecific lines affect biomass during the vegetative growth phase^9^. Therefore, of the 202 accessions, 80 with delayed flowering were excluded from the analysis.

It is also known that differences in seed size among intraspecific hybrids affect the changes in plant biomass^13^. For example, the hybrid seeds of the C24 and Col lines exhibited almost the same seed size as that of their parents, whereas the seed size of the Est line was increased compared with that of the Col line. Thus, although both the Col × C24 and Est × Col combinations showed increased biomass in their F_1_ hybrids (13.9% and 35.6% increase from the mid parental value (MPV) for Col × C24 and C24 × Col, respectively, and 37% increase from the MPV for Est × Col), the heterosis in the Est × Col hybrid can be attributed to seed size (Fig. S1). Therefore, we measured the seed areas in the remaining 122 accessions and compared them to the seed area of the control line (Col) (*P* < 0.01, Student’s *t*-test) (Fig. S2). We found that 48 accessions had a larger seed area than Col. These accessions were excluded from further analysis. After considering the genetic background and plant growth process, we finally selected 28 accessions for intraspecific hybrid evaluation (Dataset S2).

### Seed area and germination time of intraspecific F_1_ hybrids

**Figure 1:**
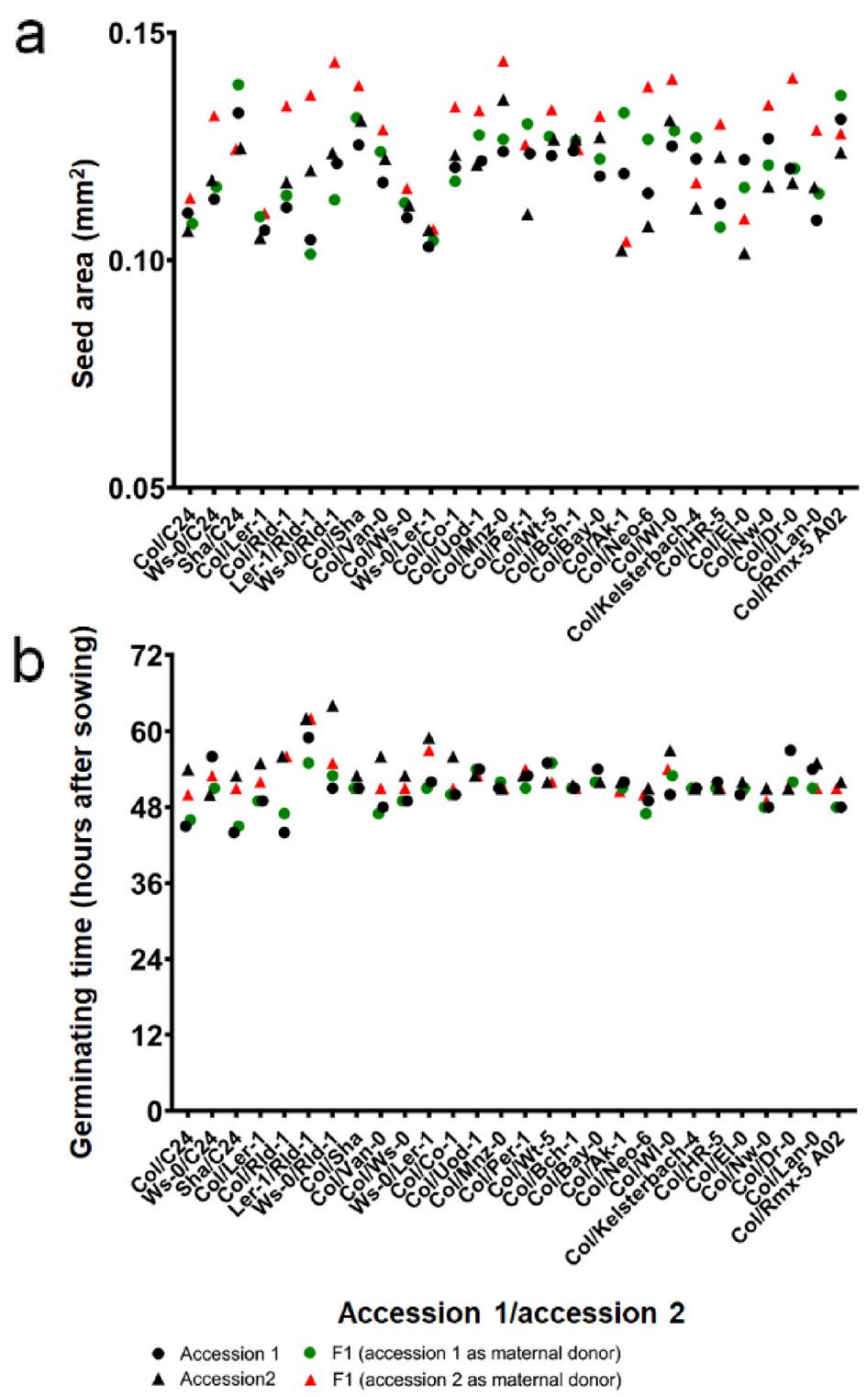
Effect of parent-origin on seed area and germinating time. (**a**) Seed area of differential parental combinations. *n* ≥ 100 seeds collected from at least three individual plants. (**b**) Germination time of intraspecific hybrids and their parents. Germinating time was calculated on the time at which 50% of cotyledons fully opened.

To investigate physiological traits and quantify biomass, seed size was first measured and compared between the hybrids and their parents. Measurements of hybrid seeds from the 28 combinations of reciprocal crosses exhibited a variation in seed area in several intraspecific hybrid combinations, suggesting that it could be attributed to maternal contribution (Figs. 1A and S3). Rld-1 × Ws-0, Rld-1 × L*er*-1, and Rld-1 × Col had a significantly increased seed area compared with the BPV (*P* < 0.01), whereas Rld-1, as the paternal donor, had the opposite effect on seed size. Similarly, the seeds of C24 × Ws-0 hybrids were notably larger than the BPV, whereas C24 × Col and C24 × Sha, with C24 as a maternal donor, were not statistically different from the BPV. Conversely, Col, Ws-0, and L*er*-1 had little effect on F_1_ seed size. Therefore, to minimize the variation in seed size between the intraspecific hybrids and their parents as much as possible, crosses with other intraspecific lines using Col as one of the parents were used in subsequent experiments.

The timing of germination, as a common physiological trait, is considered the first process of plant growth^20^. To assess differences in the timing of germination among the parental lines and the hybrids, the seed germination time was tracked (Figs. 1B and S4), and the germination timing of the F_1_ hybrids was compared with that of their parents. The results indicated that the maternal environment has a significant effect on seed dormancy and germination and that both seed size and seed germination largely reflect the effect of the maternal plants.

### Changes in heterosis levels according to a hybrid combination

**Figure 2:**
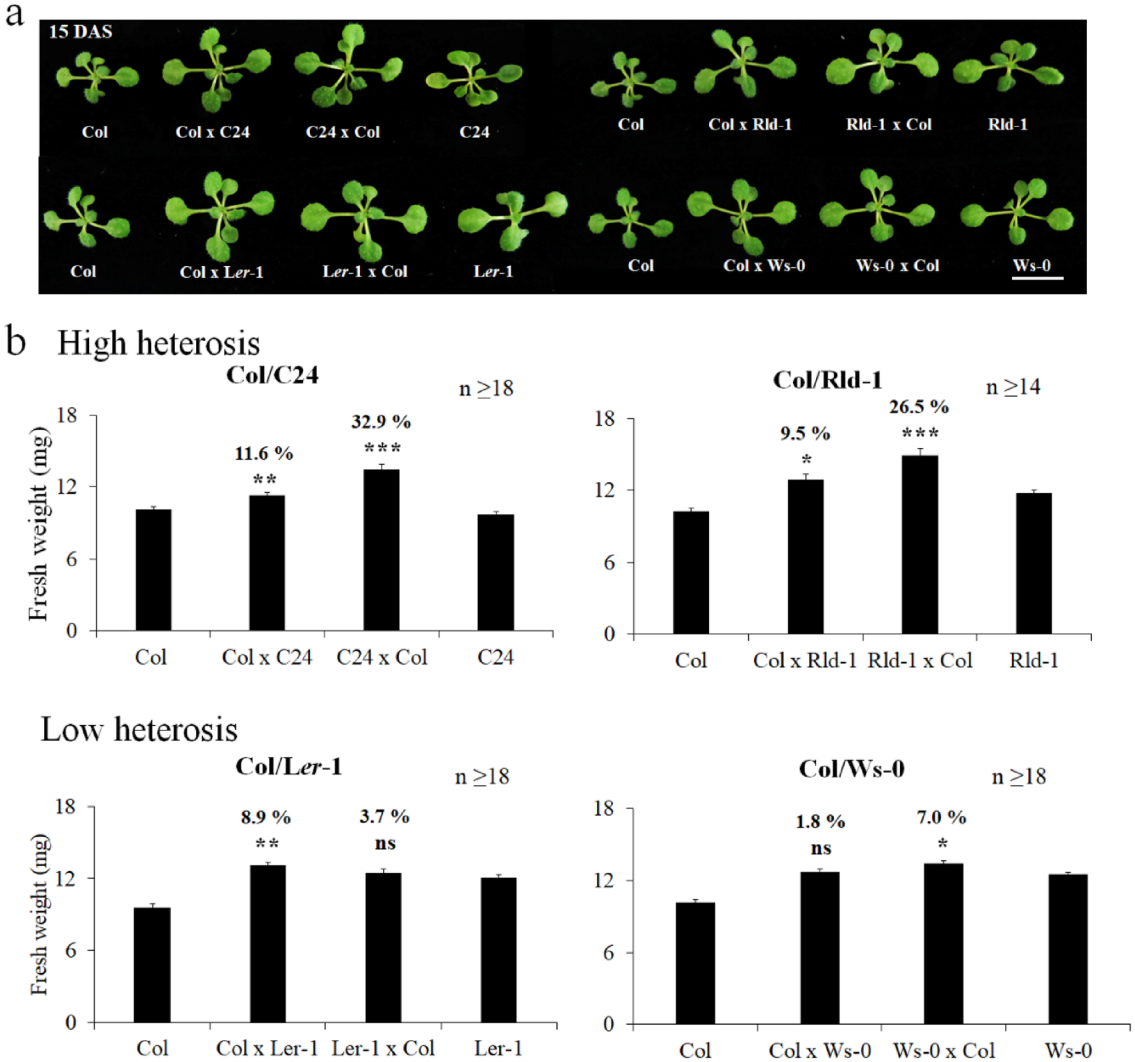
The phenotype of representative hybrids with different levels of fresh weight heterosis. (**a**) Col/C24 and Col/Rld-1 represent high heterosis levels; Col/L*er*-1 and Col/Ws-0 show low heterosis levels in 15 DAS. White bar represents 1 cm. (**b**) Levels of fresh weight heterosis of representative four hybrid combinations. Numbers above each hybrid’s bar represent a level from BPV. Black asterisks show above BPV; ns: not significant, * *P* < 0.05, ** *P* < 0.01, *** *P* < 0.001, Student's *t*-test. n represents in detail the number of plants collected from each combination. Data indicate the average and error bar (standard error).

To minimize the effects of the environmental differences and interindividual variability between the hybrids and the parental lines on biomass and developmental stages, the plants were grown under short-day and highly controlled conditions^16,19^. The parents and hybrids were adjusted to the same germination time by sowing seeds at different times based on germination time data (Figs. 2 and S5). From the agronomical point of view, hybrids need to exceed the BPV regarding the total weight to contribute to yield improvement. In addition, various time points in the developmental life cycle can modulate the differences in hybrid vigor^9^. This study focused on the early developmental stage to avoid the impact of flowering on biomass^21^. We classified heterosis according to the percentage of the fresh weight of F_1_ hybrids at 15 DAS compared with the BPV^9,13^: (1) high heterosis: two reciprocal F_1_ hybrids had significantly higher values than the BPV (*P* < 0.05), with at least one reciprocal hybrid having a 10% higher value than the BPV; (2) weak heterosis: a difference from the BPV ranging from 0 to 10%, with at least one reciprocal hybrid having a significantly higher value than the BPV; (3) no heterosis: neither of the hybrids exhibited significantly higher values than the BPV; and (4) unclassifiable combinations: hybrids obtained by reciprocal crosses that showed opposite trends.

The intraspecific hybrids generally were at the 10-leaf stage at almost 15 DAS under our experimental growth conditions (data not shown). The hybrids obtained by crossbreeding the 28 lines exhibited different levels of growth regarding fresh weight, i.e., from − 21 to 44% compared with the BPV (Fig. S5). High heterosis was detected in 9 of the 28 combinations (32% of the total hybrids). The most effective combinations were Col × Neo-6 (44% of Neo-6 × Col and 17.2% of Col × Neo-0), Col × C24 (32.9% of C24 × Col and 11.6% of Col × C24), and Col × Co-1 (36.7% of Co-1 × Col and 24.8% of Col × Co-1). Two combinations, namely, Col × L*er*-1 and Col × Ws-0, were classified into the low-heterosis class (2), with BPVs ranging from 1.8 to 8.9%. 11 of the 28 combinations did not exhibit heterosis, and the remaining six combinations showed different results in reciprocal crosses. Similarly, data from the leaf area were gathered at 5–15 DAS to support the fresh weight data (Fig. S6). It is important to note that the F_1_ hybrids obtained from the two reciprocal crosses of each combination exhibited comparable trends regarding both fresh weight and leaf area, which implies that the heterosis mechanism could also be detected by other methods. Hence, all classes, i.e., (1), (2), and (3), were considered potential candidate combinations for the extensive testing of heterosis models.

### Metabolite profiling of intraspecific *Arabidopsis* hybrids

#### Principal component analysis (PCA) of the various hybrid combinations

To identify potential metabolomics differences in biomass heterosis, hybrids of C24 and Rld-1 (high heterosis group) and L*er*-1 and Ws-0 (low-heterosis group) with Col as the common parent were analyzed for primary metabolites using gas chromatography time-of-flight mass spectrometry (GC-TOF–MS) (Fig. 2). Of the 188 peaks detected, 103 were known metabolites (Dataset S3).

**Figure 3:**
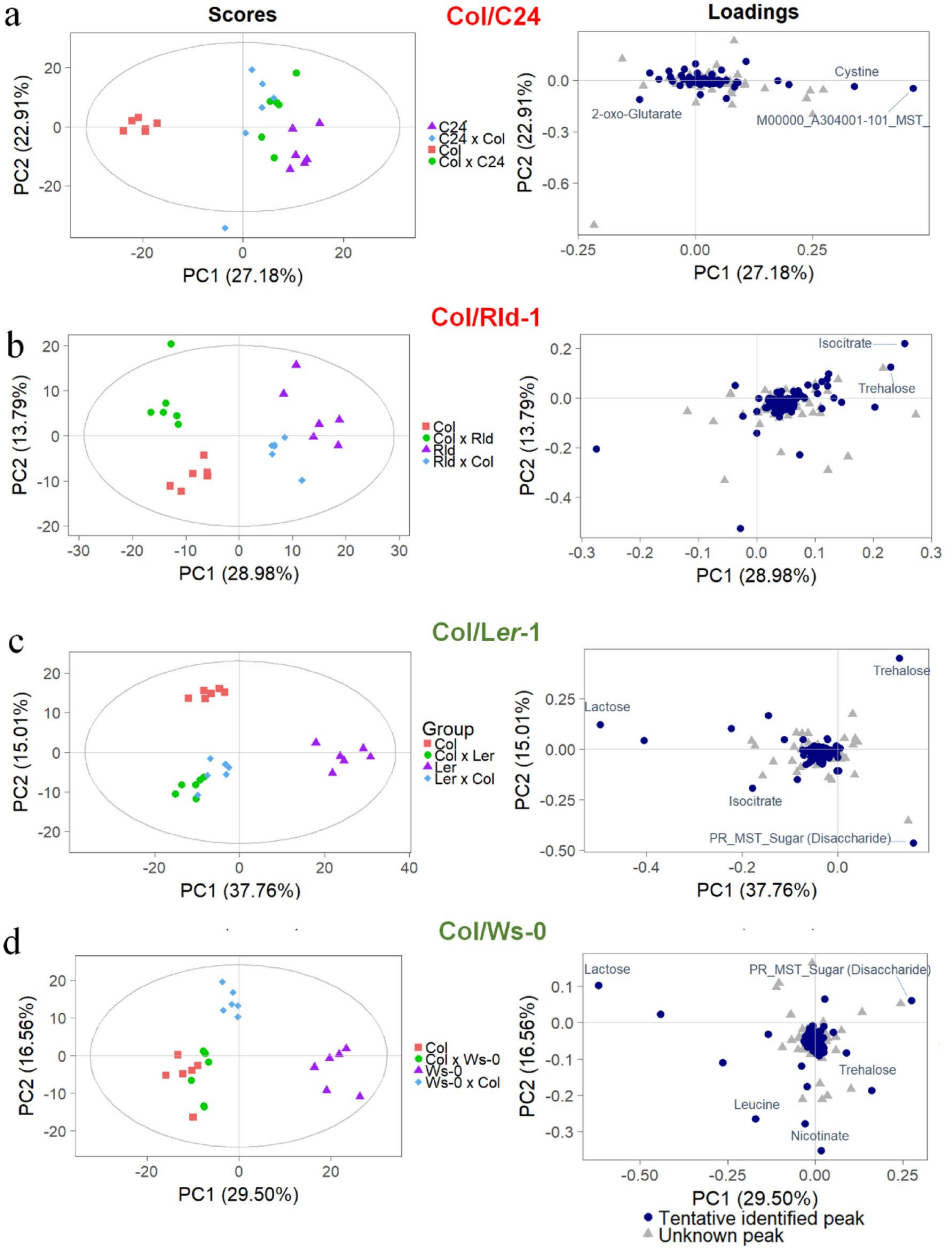
PCA plots for metabolites obtained from *Arabidopsis* combinations. Score and loading plots of (**a**) Col/C24, (**b**) Col/Rld-1, (**c**) Col/L*er*-1, and (**d**) Col/Ws-0. The log_2_ transformation of the normalized data matrix (188 peak areas × 24 leaf samples/one combination) from GC-TOF–MS was used. Red words: high biomass heterosis, green words: low-biomass heterosis.

A global analysis using PCA score plots was performed after the log_2_-transformation of the data to detect differences or similarities among the reciprocal hybrids and their parents based on non-targeted metabolite profiling (Fig. 3). Non-targeted metabolite profiling was used in this investigation to uncover all metabolites including well-known metabolites changes and unexpected metabolic responses. The score plots revealed total variability values of 50.09% (PC1: 27.17%; PC2: 22.92%) for Col × C24, 42.77% (PC1: 28.98%; PC2: 13.79%) for Col × Rld-1, 52.77% (PC1: 37.76%; PC2: 15.01%) for Col × L*er*-1, and 46.06% (PC1: 29.50%; PC2: 16.56%) for Col × Ws-0. Furthermore, the Col × C24 and C24 × Col groups were lumped together and segregated from the Col and C24 parents (Fig. 3A), similar to the Col × L*er*-1 and L*er*-1 × Col hybrids, which showed slight differences in metabolic profiles but were distinct from their parents (Fig. 3C). The Col × Rld-1 hybrids were affected by the maternal donor, and score plots tended to be closer to the maternal one (Fig. 3B). The Ws-0 × Col group was divided by both parental lines, but Col × Ws-0 and Col were close according to the PCA scores and loading plots (Fig. 3D). These results suggest that many of the candidate metabolites that may affect heterosis obtained from comprehensive metabolite profiling of different hybrid combinations were not correlated with heterosis levels and might be dependent on ecotype background. Therefore, it was necessary to focus on individual metabolites that showed differences between parents and F_1_ hybrids according to heterosis level.

### Metabolite differences among hybrids with high and low biomass heterosis

**Figure 4:**
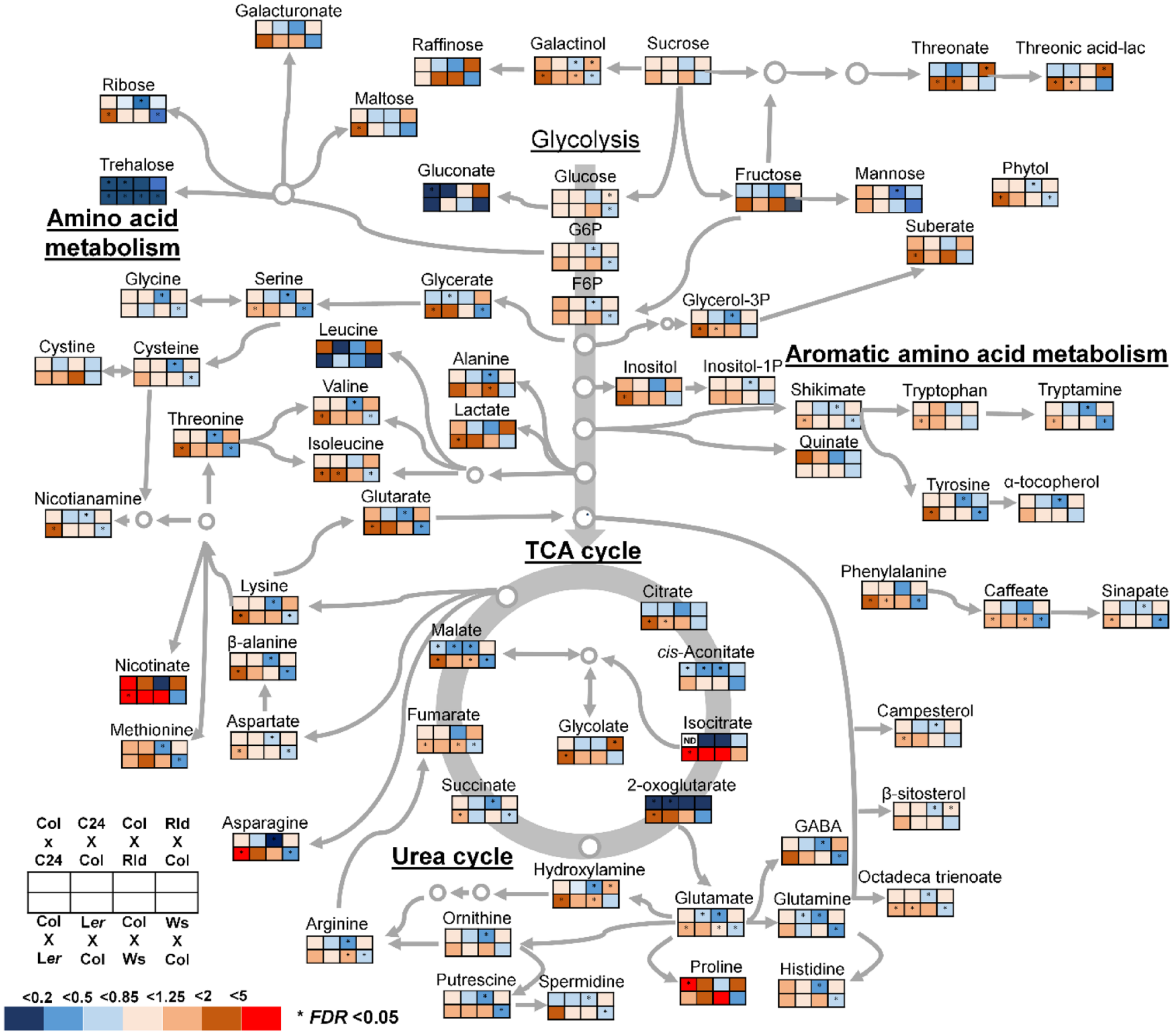
Metabolic change of F_1_ hybrid to their parents. The fold change of the F_1_ hybrid’s metabolic change is normalized concerning the mid-parent value. Fold change is visualized by color; red, upregulated in F_1_ hybrid; blue, downregulated in F_1_ hybrid. ND not detected. * *FDR* < 0.05, significant increase/decrease of F_1_ hybrid as compared with each parent, based on multiple* t*-tests.

The differences in metabolite profiling between the hybrids and their parents were analyzed using multiple *t*-tests (*FDR* < 0.05). Metabolites that were significantly increased or decreased in the comparison of F_1_ individuals with their parents are shown in the heat map (Fig. S7). The full dataset used in this heat map is presented in Dataset S4. The pattern of the significant metabolite changes between the hybrid and the parental lines for the Col × Rld-1 and Col × Ws-0 hybrids showed an opposite trend compared with the hybrids obtained by reciprocal crosses. Conversely, the pattern of the significant metabolite changes between the hybrids and the parental lines for Col × C24, as well as Col × L*er*-1, showed a similar trend compared with the hybrids obtained by reciprocal crosses (Fig. S7, Dataset S3). Notably, the lowest number of metabolites showing significant changes between the hybrids and the parental lines was lowest in crosses between Col and C24, which showed high heterosis (Fig. S7, Dataset S3). Figure 4 presents the metabolic map of the central metabolic pathways of F_1_ hybrids with their mid-parent value (MPV). The common sugars and sugar derivatives were mostly unaltered in the F_1_ hybrids, and the levels of sucrose, maltose, raffinose, glucose, and ribose showed no clear trend between F_1_ hybrids with high and/or low heterosis. Previously, Groszmann et al. reported that sugar transporter-related genes, including *STP1*, *STP14*, *ERD6*, and *PLT6*, are upregulated in all three hybrids (i.e., C24 × L*er*, C24 × Col, and Col × L*er*)^22^. In our transcriptome data, no difference was found in the expression levels of *STP1*, *STP14*, *ERD6*, and *PLT6* in both Col × C24 and C24 × Col hybrids (Fig. S8). Starch content is also compared in hybrids and their parents to identify effects on hybrid vigor. Hybrids of Col × C24, Col × Ws-0, and L*er*-1 × Ws-0 were selected as representatives of high heterosis, low heterosis, and no heterosis, respectively, to analyze the amounts of starch. Results showed that reciprocal hybrids had starch levels comparable to their parental lines at all heterosis levels during the mid-light phase (ZT6) and the late-light phase (ZT11) (Fig. S9). In addition, our metabolome data suggested that amino acid levels exhibited unclear trends, whereas the main metabolic changes detected between the F_1_ hybrids and their parents pertained to TCA cycle intermediates, citrate, cis-aconitate, isocitrate, and 2-oxoglutarate, which were downregulated in the F_1_ hybrids with high heterosis (Fig. 4). Therefore, we decided to focus on TCA cycle intermediates to examine their involvement in heterosis.

**Figure 5:**
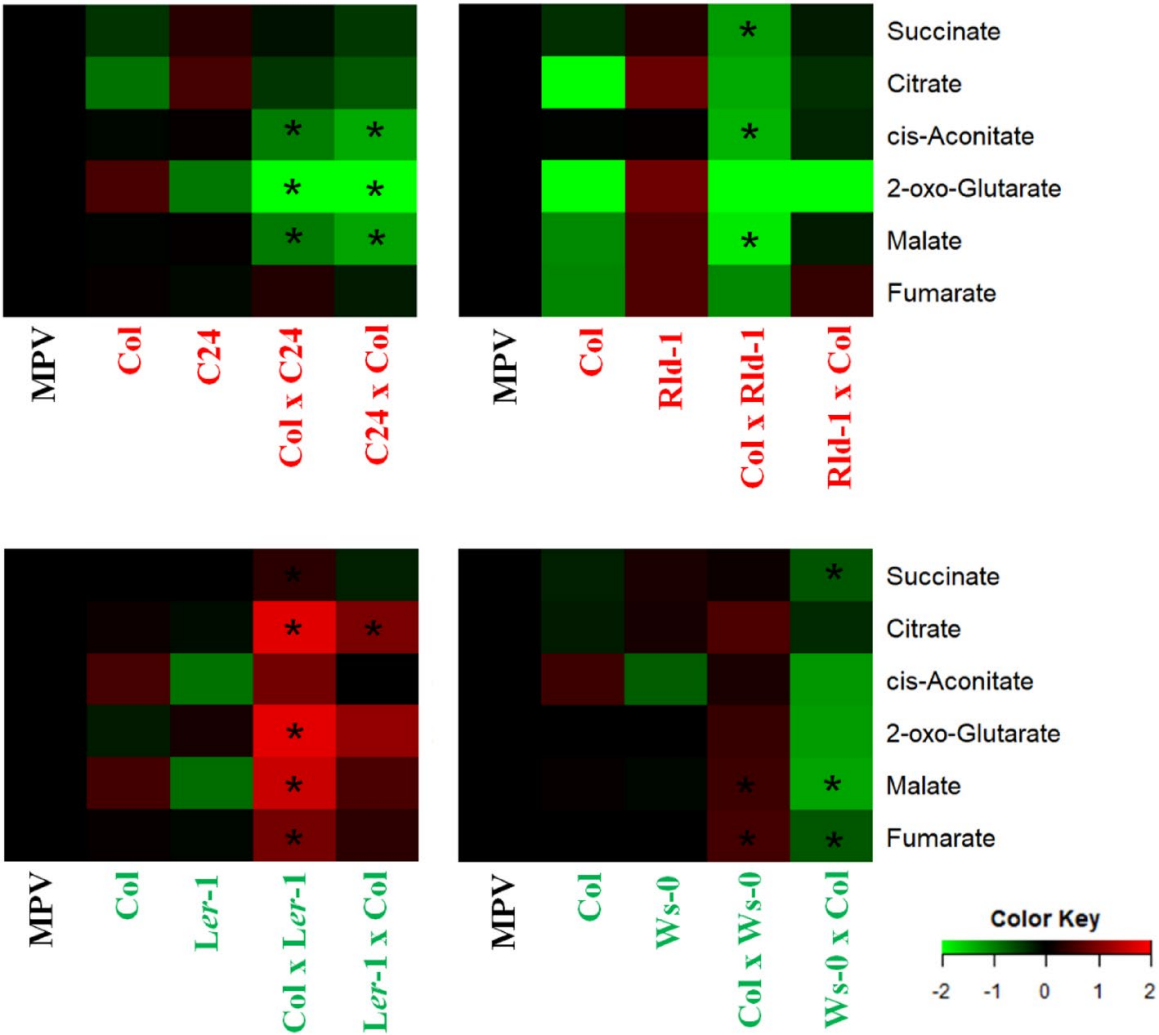
Levels of TCA cycle intermediates in hybrid combinations. Asterisks mean a significant difference by multiple* t*-tests (*FDR* < 0.05), increased/decreased metabolites compared with both parents. Differences in red/green colors exhibit the log_2_ fold change (up/down) normalized to MPV (MPV: control = 0 in log_2_ fold change). Red/green words mean the name of high/low-biomass heterosis.

**Figure 6:**
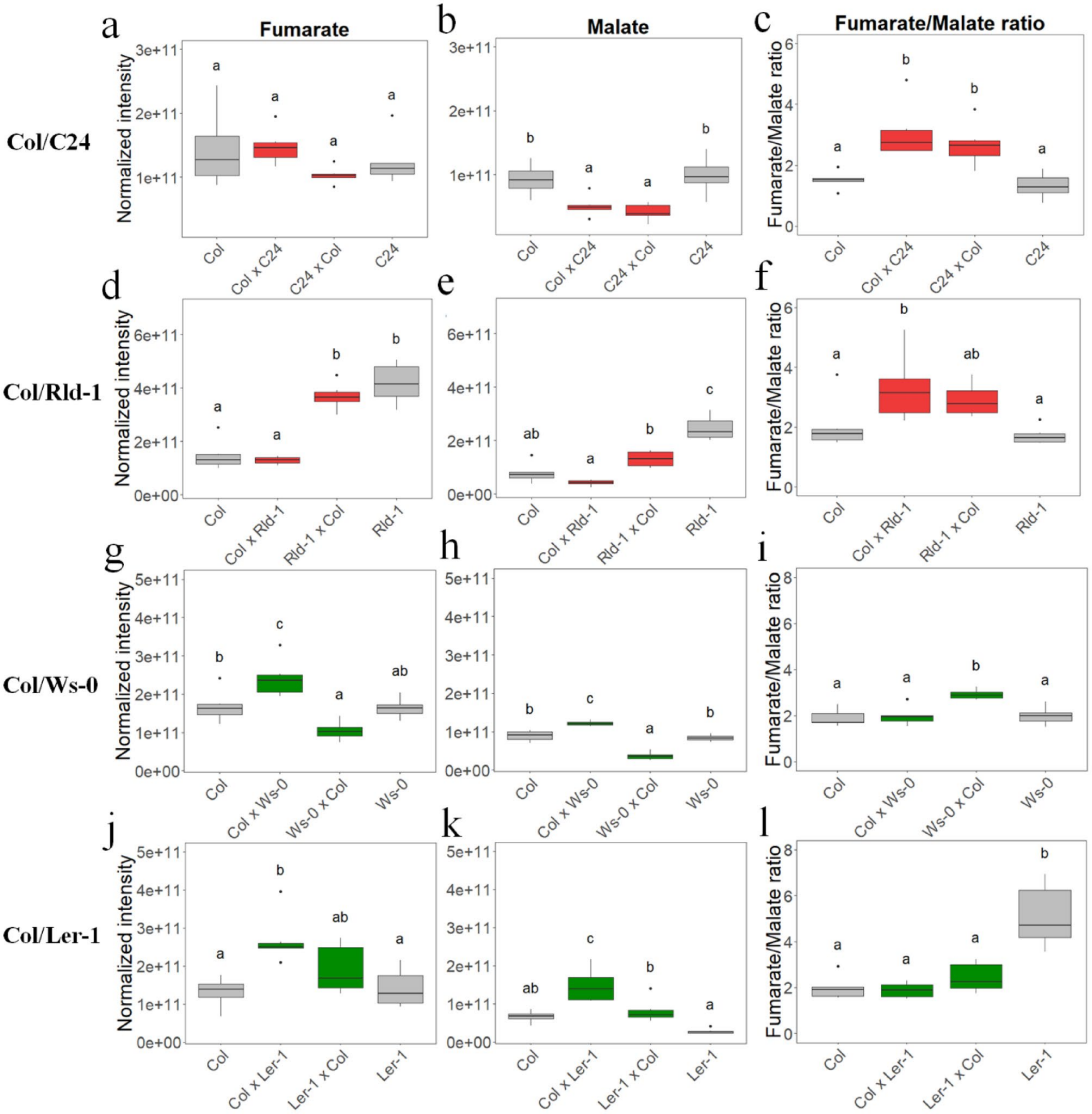
Analysis of fumarate, malate, and fumarate/malate ratio in Col/C24, Col/Rld-1, Col/Ws-0, and Col/L*er*-1. Col/C24: fumarate (**a**), malate (**b**), and fumarate/malate ratio (**c**). Col/Rld-1: fumarate (**d**), malate (**e**), and fumarate/malate ratio (**f**). Col/Ws-0: fumarate (**g**), malate (**h**), and fumarate/malate ratio (**i**). Col/L*er*-1: fumarate (**j**), malate (**k**), and fumarate/malate ratio (**l**). Box plots present median value and upper and lower quartiles. Difference letters over box plots are displayed significant value according Tukey’s test, *P* < 0.05.

TCA cycle intermediates are the principal components responsible for driving biomass, as reported previously^14,17^. Therefore, we focused on the metabolites in the TCA cycle in several hybrid combinations. We found a decreasing trend in Col × C24 and Col × Rld-1, with higher levels of heterosis (Figs. 4 and 5). The three intermediates of the TCA cycle, namely, malate, 2-oxoglutarate, and aconitate, were detected at lower levels in high heterosis hybrids, i.e., Col × C24, Col × Rld, and Ws-0 × Col, compared with the MPV (Figs. 4 and 5). By contrast, the low-heterosis Col × L*er*- and Col × Ws-0 hybrids showed higher levels than the MPV (Figs. 4 and 5). The fumarate/malate ratios of the Col × C24, C24 × Col, Col × Rld, Rld × Col, and Ws × Col hybrids were increased by 2.2-, 1.9-, 1.8-, 1.5-, and 1.5-fold, respectively, compared with the MPV (Fig. 6). Malate has a greater pKa value than fumarate, and a higher fumarate/malate ratio offers a beneficial mechanism for the pH-mediated modulation of intracellular flow and biomass^23^. These results suggest that the components of the TCA cycle play an important role in the mechanism of heterosis.

To understand the expression levels of TCA cycle-related genes, we performed RNA sequencing in the hybrids and parental lines for Col × C24. One-way ANOVA revealed that only two genes (i.e., *FUM2* and *ATACA2*) showed a significantly differential expression pattern among the hybrids and parents among 78 TCA cycle-related genes based on MapMan annotation^24^ (Fig. S10a and Dataset S5). The differential expression pattern of the two genes was mainly based on differences between the two parents rather than on F_1_-hybrid-specific regulation (Fig. S10b). Although the RNA sequencing data of Col × C24 did not reveal any F_1_-hybrid-specific expression pattern for the TCA cycle-related genes, we also performed a quantitative PCR analysis of *FUM1* and *FUM2* in the Col × C24, Col × Rld-1, Col × Ws-0, and Col × L*er*-1 hybrids because it was previously reported that fumarase determines the fumarate/malate ratios^18^. As shown in Fig. S11, the expression levels of *FUM1* and *FUM2* were not significantly different between the two hybrids and their BPVs. This indicates that the high fumarate/malate ratio observed in high heterosis hybrids was not caused by changes in transcriptional regulation. Post-transcriptional or post-translational regulation may correlate with changes in the metabolites of the TCA cycle. Changes in the fumarate/malate ratio could also be caused by increased metabolic activity in the F_1_ hybrids.

## Discussion

The effect of parental origin on seed size has been recognized^9,25^. This study examined several F_1_ hybrids that develop larger mature embryos compared with their parental lines. Hybrids of C24 and L*er*-1 have been suggested to promote or suppress embryo heterosis^9^. We found that the use of the Rld-1 and C24 accessions as maternal donors increased F_1_ seed size (Figs. 1 and S3). Thus, for some of the F_1_ hybrid combinations examined here, it is possible that F_1_-hybrid-specific modifications based on maternal lineage traits caused the increase in seed size. A possible explanation for this observation is that the embryos of F_1_ hybrid seeds obtained by reciprocal crosses are epigenetically different even though they are genetically identical. In addition, the composition of the seed coat is affected by the maternal origin, suggesting that the contribution from the two parents is unequal. It is also possible that the maternal-derived organelle genome (chloroplast and mitochondrial DNA), which is important for seed size heterosis, exhibited different interactions with the nuclear genome in the two parents compared with the hybrid lines^26,27^.

C24 × L*er* hybrids exhibit a high level of heterosis regarding biomass production in *Arabidopsis*^28–30^, with upregulation of photosynthesis-related genes and early emergence of new leaves^28^. The seeds of reciprocal C24 × L*er* hybrids germinated 7–12 h earlier than did their parents^29^. However, when the germination times of the parental and F_1_ hybrids were matched, the chloroplast-related genes of F_1_ hybrids were not downregulated or changed compared with their parents^31^, suggesting that the changes detected in C24 × L*er* hybrids may be attributed to differences in developmental stages between the hybrids and their parents. This is a potential explanation for the differences observed in the developmental stages of the hybrids and their parents. Therefore, in this study, the germination times were identical among the lines to eliminate the effect of the differences in developmental stages. In addition, early germination is considered an indicator of a heterotic phenotype^29^. However, in our data from multiple intraspecific hybrids, none of the nine combinations that exhibited high heterosis in seedlings showed early germination in hybrids (Figs. 1 and S4). Thus, although an early germination time may contribute to a higher biomass^28,29^, it does not necessarily seem to be an indicator of heterosis in *Arabidopsis*.

Carbohydrates or sugars are a primary source of carbon and energy and play a fundamental role in plant growth regulation^32–34^. *Arabidopsis* allotetraploids were reported to contain more starch and sugars, as well as increased biomass, with almost 12% more chlorophyll and 10% more starch than the BPV^35^. Using a set of recombinant inbred lines from a cross of Col-0 and C24 ecotype, carbohydrates or sugars-related metabolites such as glucose-6-phosphate, fructose-6-phosphate, or sucrose have significantly correlated to biomass^14^. Therefore, we assessed whether sugar derivatives were associated with the heterotic characteristics in several F_1_ hybrids. In this study, the hybrids were grown under short-day conditions without sucrose added to the medium, thus limiting the carbon source required for growth. We hypothesized that if sucrose is involved in the heterosis mechanism, then differences in sugar-induced carbohydrate metabolic pathways in hybrids should emerge. Sucrose may play a role in the induction of faster growth rates, especially during early development. In our study, trehalose tended to be lower in all F_1_ hybrids compared with inbred lines under short-day conditions without sucrose (Dataset S3). However, in our study, there were no clear differences in the overall carbohydrate metabolic pathway and no changes in metabolites at different levels of heterosis. In addition, the sugar-transport-responsive *STP1*, *STP14*, *ERD6*, and *PLT6* genes were upregulated in the C24 × L*er*, C24 × Col, and Col × L*er* hybrids^22^. Nevertheless, our transcriptome data showed no significant upregulation of these genes in Col × C24 and C24 × Col compared with their parents (Fig. S8). Moreover, starch metabolism is essential for growth^36^. Therefore, we also examined the starch accumulation in the hybrids of Col × C24 (high heterosis), Col × Ws-0 (low heterosis), and Ws-0 × L*er*-1 no heterosis) to determine whether starch contributed to heterosis. Under both ZT6 and ZT11 tested, the reciprocal hybrids at all heterosis classes had starch levels no greater than the parental lines (Fig. S9). These results suggest that the carbohydrate metabolite profiling and starch are not altered when biomass heterosis occurs in the F_1_ hybrids of *Arabidopsis* at least 15DAS young growth stage under short-day growing conditions.

In oxygen metabolism, the TCA cycle is essential for large energy intermediates^17^. Also, regarding the close association between biomass increase and metabolic composition, it has been reported that central metabolic pathways, i.e., the TCA cycle and amino acid pathway, are negatively correlated with biomass gain^14^. A QTL analysis in recombinant inbred lines (RILs) and the closely related species Col × C24 and C24 × Col revealed that *FUM2* was a candidate gene for heterosis^37^. Recently, associations between heterotic growth traits and fumarate and malate levels, as well as high fumarate/malate ratios, were detected in large populations of *Arabidopsis* accessions, RILs, near-isogenic lines, hybrids, and different photoperiods^18,19^. In this study, several intraspecific hybrids were used to identify metabolites associated with growth. As a result, we observed higher fumarate/malate ratios in hybrids showing high heterosis levels, similar to that reported previously (Fig. 6). Thus, the TCA cycle appears to be a core factor associated with vigorous growth. In oxygen metabolism, the TCA cycle is essential for large energy intermediates^17^. Fumaric acid has a lower pK_a_ value than that malic acid, and a higher fumarate/malate ratio provides an advantageous mechanism for the pH-mediated regulation of intracellular flux and biomass^23^. In *Asparagus sprengeri*, a decrease in cytosolic pH is associated with γ-aminobutyrate (GABA) synthesis (glutamate + H^+^ ≥ GABA + CO_2_)^38^. The *fum2* knock-down mutant exhibited a higher malate content, whereas the fumarate content remained low, and a decrease in biomass was detected under high-nitrogen conditions. This indicates that nitrate is not a major source of ammonium and that fumarate and malate help in maintaining the pH as nitrate is converted to ammonium^23^. The final core function of the TCA process is the production of energy^39^. Moreover, evidence has emerged in support of higher TCA cycle fluxes, photosynthetic capacity, and crop yields afforded by stomatal opening and closing movements^40–42^. At the protein level, enzymes that participate in the TCA and Calvin–Benson cycles were broadly upregulated, indicating an increase in the flux to provide more energy for enhancing growth at longer photoperiods^43^. The details of the upregulation of photosynthesis by controlling fumarate and malate concentrations remain unclear^40,42^. In this report, the TCA cycle may have been regulated in the parental accessions, thus limiting the energy flux necessary for growth. In the case of high heterosis, hybrids can accelerate the TCA flux and can produce more energy as biomass. Previously, reduced levels of TCA cycle intermediates were shown to result in enhanced rates of photosynthesis and aerial growth throughout carbon flux^14,44^. This negative correlation seems to indicate that strong growth is triggered to diminish the levels of central metabolites rather than an increase in the supply of the components used for cellular synthesis^14^. These examples reveal the importance of mitochondrial reactions for cellular function and, hence, for higher biomass in plants. Therefore, we hypothesized that photosynthesis and/or nitrogen metabolism occur when heterosis has a high TCA cycle flux. Because TCA cycle-related genes do not exhibit any significant changes in their transcriptional levels, we assumed that the reduced levels observed in TCA cycle intermediates occurred at the post-transcriptional level or were caused by the action of specific regulatory factors. The detailed effects of these regulatory mechanisms involved in such interactions remain to be studied.

In conclusion, we report here that general physiological traits, such as seed area and germination time, were affected by the maternal donor. In addition, the minimization of environmental differences and interindividual variability, which affect biomass and developmental stages, led to various levels of heterosis and provided a taxonomy of combinations for further study. The metabolomics analysis of intraspecific *Arabidopsis thaliana* revealed that TCA fluxes seem to be involved in the acquisition of heterotic phenotypes. This will provide insight into the phenomena of heterosis and higher plant growth.

## Materials and methods

### Plant material and growth conditions

A total of 202 accessions obtained from the *Arabidopsis* Biological Resource Center (the Ohio State University, U.S.) used in this study (Dataset S1) were planted at Tsukuba-Plant Innovation Research Center, University of Tsukuba, Tsukuba (36° 11′ N and 140° 10′  E) in 2017–2019. The experimental study of the plants, including the collection of plant material, complied with relevant institutional, national, and international guidelines and legislation. Seeds of both F_1_ hybrids and parental controls were generated by hand pollination as reference^13^ and were collected at a comparable time.

Seeds were sterilized, stored at 4 °C for three days, and then sown on square dishes containing 1/2 Murashige and Skoog (MS) salts with vitamins, pH 5.7, 1% (w/v) agar without sucrose. Plates were divided into four compartments comprising of two parental lines and their hybrids and then replaced in developmental chambers under short-day conditions (12 h in light/12 h in the dark) at 22 °C, fluxes of 120 µmol photons m^2^ s^−1^. 5 DAS seedlings were transferred to 150 mm-diameter plates and developed under the same conditions. The dishes were rotated around the development chamber daily to ensure uniform lighting conditions^16^.

For seed germinating time, the complete opening of the cotyledons was recorded every 12 h after sowing (HAS) until 72 HAS. Data were collected every 4 h from 48 to 60 HAS as green cotyledons appeared within this period. The time of germination was defined as the time when 50% of the seed had fully opened cotyledons. Based on these data, the seed sowing times of the phenotypic experiments were matched to eliminate differences in germination time between the parents and their reciprocal hybrids.

### Measurement of seed area, leaf area, and shoot fresh weight

After harvest, seed images were recorded by VHX-700F digital microscopy (KEYENCE, Japan), and their areas were measured with ImageJ software^45^. At least five individual plants were measured in each line. Leaf area and fresh weight data were collected as described^19^. Using ImageJ software, the leaf area of seedlings from 5 to 15 DAS was calculated^44^. Data on leaf numbers were collected by counting all visible leaves. Each plant’s seedlings were weighed when they had 10 leaves and 15 DAS on them, which is known as their fresh weight.

The mid-parent value (MPV) was calculated as the average of the trait value of the two parents. The better-parent value (BPV) was defined as the trait value of the better-performing parent in the cross.

### Metabolite data with GC-TOF–MS

Samples for GC-TOF–MS analysis were collected at zeitgeber time (ZT) = 6, along with documentation of shoot fresh weight at 15 DAS. The entire rosettes were harvested in 6 replicates per line at the same 10-leaf number stage, each replicate comprising two bulked plants; then, they were promptly frozen in liquid nitrogen and kept at -80 °C until extraction preparation.

Extraction of each sample was done by mixing each sample with a ratio of 25 mg of tissues (fresh weight) per 01 μl of extraction medium (methanol/chloroform/water [3:1:1 v/v/v]) containing 10 stable isotope reference compounds (Cambridge Isotope Laboratories, USA; Spectra Stable Isotopes, USA and C/D/N ISOTOPES, Canada) using a Retsch mixer mill MM310 at a frequency of 30 Hz for 3 min at 4 °C. Each isotope was adjusted to the final concentration of 22.5 ng/μl/injection. After centrifugated for 5 min at 15,100×*g*, 400 μl of the supernatant was used for further analysis. The extracts were dried in a Savant SPD2010 SpeedVac Concentrator (Thermo Electron Corporation, Waltham, MA, USA).

Each sample was then added with 20 μl of methoxyamine hydrochloride (20 mg/ml in pyridine) at room temperature for methoximation. After 30 h of derivatization, the sample was trimethylsilylated by shaking with 20 μl of *N*-methyl-*N*-trimethylsilyltrifluoroacetamide with 1% trimethylchlorosilane (Pierce, USA) at 37 °C in 1 h, then added with 20 μl of n-heptane. In the vacuum glove box, VSC-100 (Sanplatec, Japan) was packed with 99.9995% (G3 grade) dry nitrogen, and all derivatization steps were carried out. The analysis of metabolites by GC-TOF/MS has been described previously^46^.

### Quantification of starch

Starch quantification was performed as previously described^47^, with slight modifications. Seedlings were grown for 15 days in short-day conditions, and then the whole rosettes were harvested at ZT6 and ZT11 and weighed. Harvested rosettes were immediately frozen in liquid nitrogen, and two or three plants bulked to generate three replicates. Firstly, plant materials were ground and suspended in 1 ml 0.7 M ice-cold perchloric acid. Then, the soluble fractions were separated and removed by centrifugation (15,300 g, 15 min, 4 °C). The insoluble fractions were rinsed twice with water and washed out the chlorophyll with 80% (v/v) ethanol heated to 50 °C five times at least. The pellet containing starch was rinsed twice with water and resuspended in 1.2 ml of water. 500 μl of resuspension was dispensed to every two tubes, and one tube was used as blank. Reaction buffer and thermostable α-amylase were added and heated to 100 °C for 15 min, and 10 μl of α-amyloglucosidase solution was added when the mixture cooled to 50 °C using a heat block for 5 min and incubated for 30 min. Finally, the starch was quantified by measuring the product of D-glucose using the glucose oxidase/peroxidase (GOPOD) method.

### Statistical analysis

Student’s* t*-test (*P* < 0.05) was used to compare phenotypic data. For the metabolic analysis, all variables were transformed (log_2_); then, multiple *t*-tests were performed with a false discovery rate (FDR) approach and a desired FDR of < 0.05^48^. Box plot visualization and statistical analysis above box plots (Tukey’s test, *P* < 0.05) were performed using R software. Principal component analysis (PCA) was used in R software along with pcaMethods and ggplot2 to display changes in the levels of 188 metabolites discovered by GC-TOF–MS. For the transcriptome analysis, one way-ANOVA was performed with an FDR approach and a desired FDR of < 0.05. Bar plot visualization and statistical analysis above bar plots (Tukey's test, *P* < 0.05) were performed using R software.

### Expression analysis

15 DAS *Arabidopsis* seedlings from Col, C24, Rld-1, Ws-0, L*er*-1, and their reciprocal F_1_ hybrids harvested at ZT6 were used as samples for RNA expression profiling. The RNA extraction, cDNA synthesis, and quantitative real-time polymerase chain reaction were performed as previously described^19^. Gene-specific primer sequences were as follows: *FUM1* (AT2G47510) (5′–TGCGTTATGCCACCTCTCTG–3′ and 5′–TTGCTTTCCCAATCGTCGGA–3′) and *FUM2* (AT5G50950) (5′–GTCGCATGTACTCTACCCCG–3′ and 5′–GTGCAGCCAGAGCTTCAAAC–3′). Expressions of these target genes were normalized using *AT5G12240* as a control gene^49^. RNA-seq was performed for the F_1_ progeny of reciprocal crosses between the Col-0, C24, and their reciprocal F_1_ hybrids of 15 DAS seedlings. Sample preparation, sequencing, and data analyses by MinION (Oxford Nanopore Technologies, UK) were performed as previously^50^.

## Supplementary Information

Supplementary Figures.

Supplementary Information 1.

## Data Availability

The raw sequence reads we generated in this study have been deposited at DDBJ Sequenced Read Archive under accession numbers DRX409928—DRX409939 of PRJDB14952.
