# Supplementary material for: Morphological and metabolomics profiling of intraspecific Arabidopsis hybrids in relation to biomass heterosis
Source: Sci Rep. 2023 Jun 12;13:9529. doi: 10.1038/s41598-023-36618-y (PMC10261038; doi:10.1038/s41598-023-36618-y)
Supplement: Supplementary file 1 — Supplementary Figures. [file 41598_2023_36618_MOESM1_ESM.pdf]

Supplementary Figure

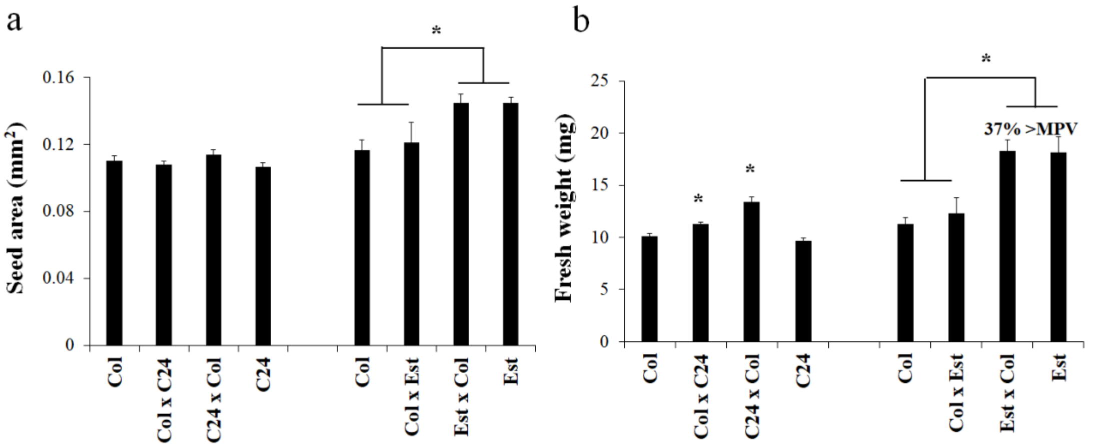

Figure S1. Seed area and fresh weight of C24 and Est crossed to Col. (a) Seed area and (b) fresh weight of Col/C24 and Col/Est. Seed area data were an average of at least 100 seeds collected from at least five individual plants ± SE. Fresh weight data was collected in 15 DAS ($n \geq 12$), mg/plant ± SE; SE, standard error. Significant value compared with BPV in Col/C24 combination and grouped comparison on Col/Est combination (*$P < 0.001$ by Student's *t*-test).

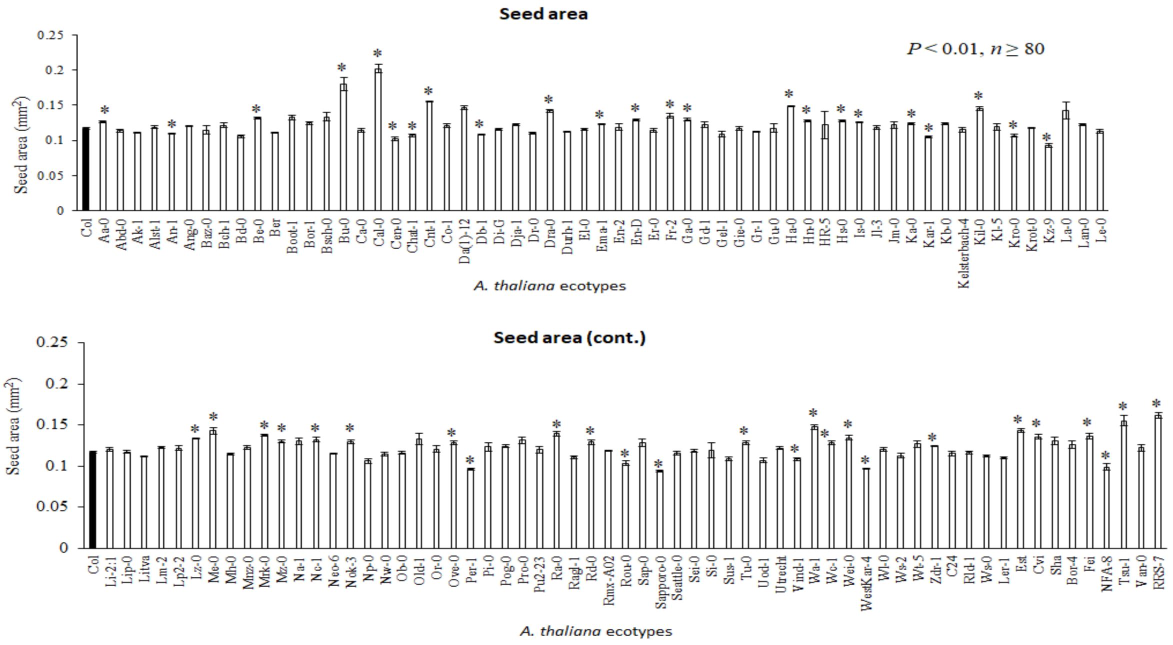

Figure S2. Seed area of Arabidopsis accessions.

Data were average ± SE; SE, standard error ($n \geq 80$ seeds of three individuals). The black bar is data of Col as the control line for the comparison test (*, $P < 0.01$ by Student's *t*-test).

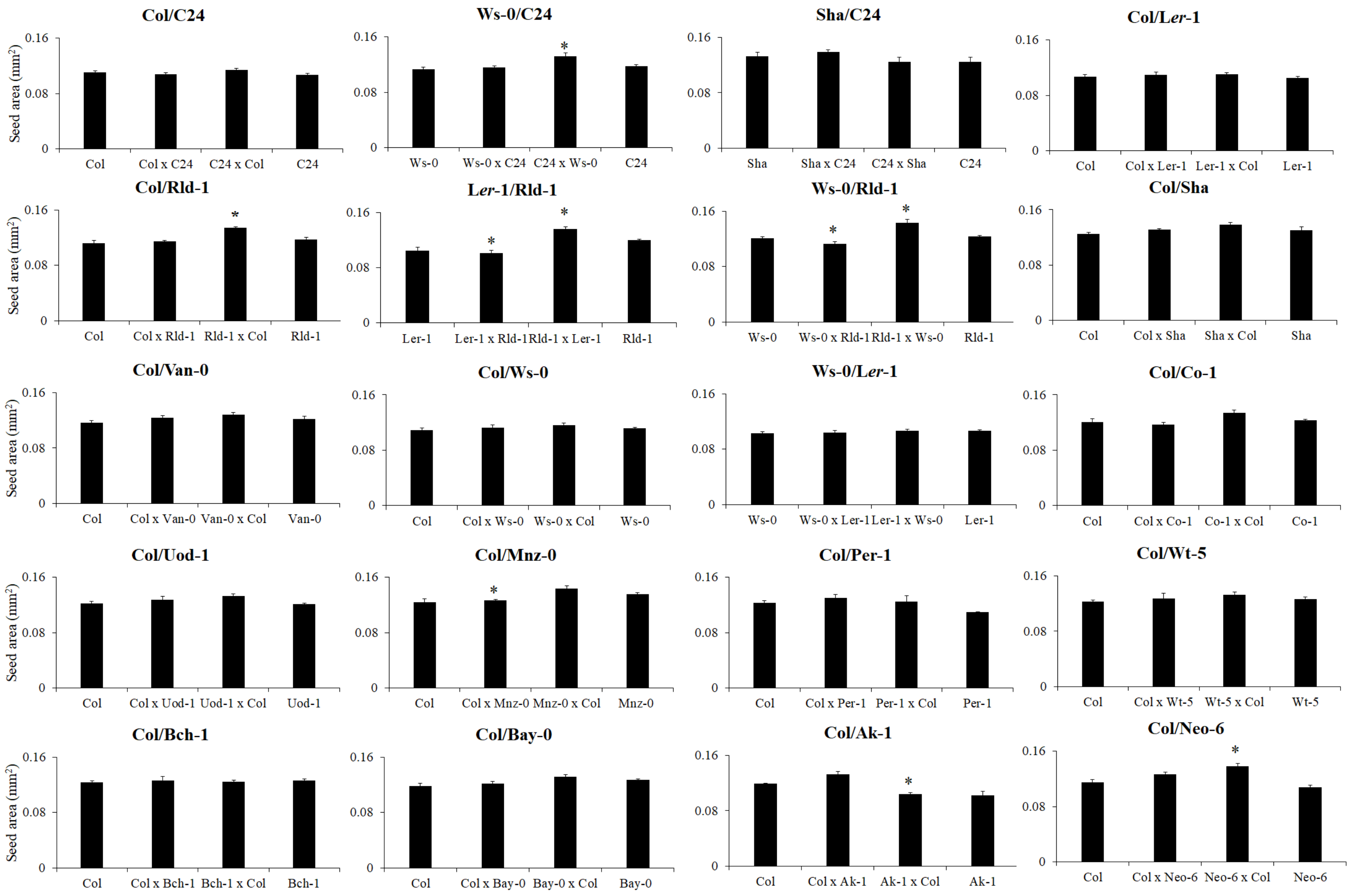
Col/C24
Seed area (mm2)
0.16
0.08
0
Col
Col x C24
C24 x Col
C24
Ws-0/C24
Ws-0
Ws-0 x C24
C24 x Ws-0
C24
Sha/C24
Sha
Sha x C24
C24 x Sha
C24
Col/Ler-1
Col
Col x Ler-1
Ler-1 x Col
Ler-1
Col/Rld-1
Col
Col x Rld-1
Rld-1 x Col
Rld-1
Ler-1/Rld-1
Ler-1
Ler-1 x Rld-1
Rld-1 x Ler-1
Rld-1
Ws-0/Rld-1
Ws-0
Ws-0 x Rld-1
Rld-1 x Ws-0
Rld-1
Col/Sha
Col
Col x Sha
Sha x Col
Sha
Col/Van-0
Col
Col x Van-0
Van-0 x Col
Van-0
Col/Ws-0
Col
Col x Ws-0
Ws-0 x Col
Ws-0
Ws-0/Ler-1
Ws-0
Ws-0 x Ler-1
Ler-1 x Ws-0
Ler-1
Col/Co-1
Col
Col x Co-1
Co-1 x Col
Co-1
Col/Uod-1
Col
Col x Uod-1
Uod-1 x Col
Uod-1
Col/Mnz-0
Col
Col x Mnz-0
Mnz-0 x Col
Mnz-0
Col/Per-1
Col
Col x Per-1
Per-1 x Col
Per-1
Col/Wt-5
Col
Col x Wt-5
Wt-5 x Col
Wt-5
Col/Bch-1
Col
Col x Bch-1
Bch-1 x Col
Bch-1
Col/Bay-0
Col
Col x Bay-0
Bay-0 x Col
Bay-0
Col/Ak-1
Col
Col x Ak-1
Ak-1 x Col
Ak-1
Col/Neo-6
Col
Col x Neo-6
Neo-6 x Col
Neo-6

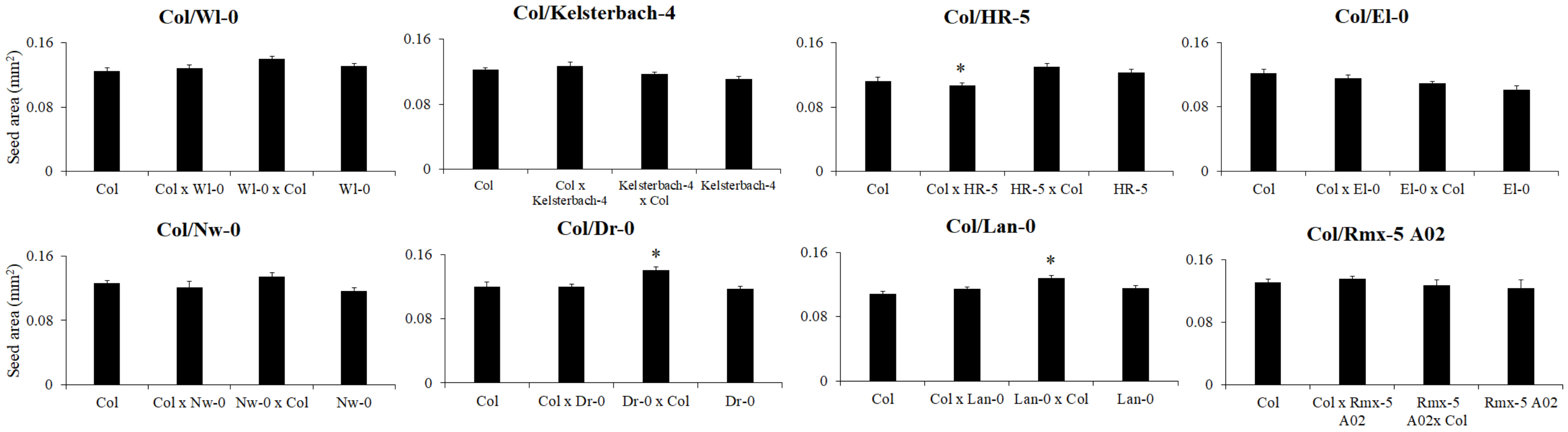

Figure S3. Measurements of seed area of differential parental combinations.

Statistical comparisons between two reciprocal hybrids and their BPV with $P < 0.01$ (Student's t-test), $n \geq 100$ seeds collected at least three individual plants. All error bars are standard errors.

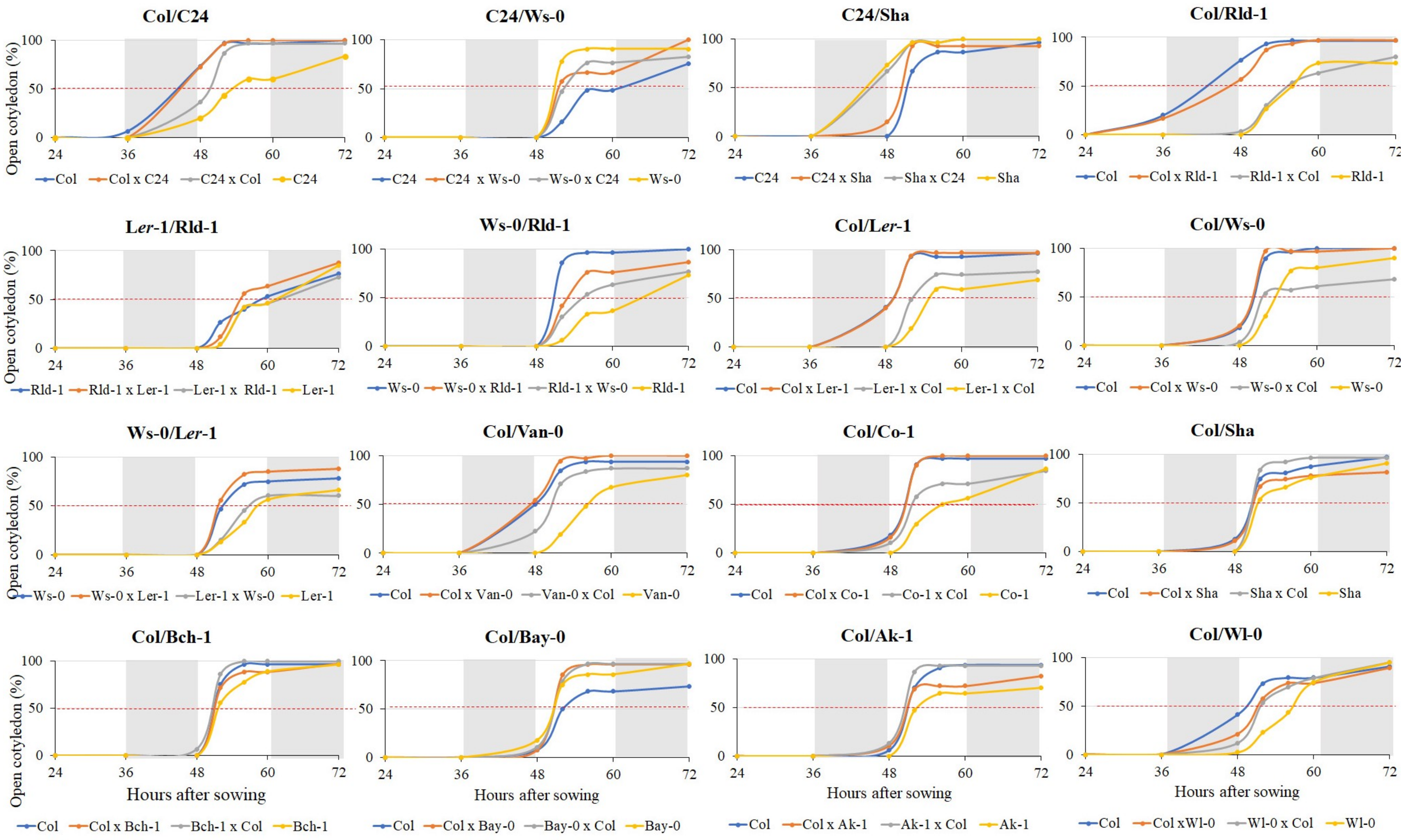

Col/C24
C24/Ws-0
C24/Sha
Col/Rld-1
Open cotyledon (%)
100
50
0
24
36
48
60
72
Col
Col x C24
C24 x Col
C24
C24
C24 x Ws-0
Ws-0 x C24
Ws-0
C24
C24 x Sha
Sha x C24
Sha
Col
Col x Rld-1
Rld-1 x Col
Rld-1
Ler-1/Rld-1
Ws-0/Rld-1
Col/Ler-1
Col/Ws-0
Rld-1
Rld-1 x Ler-1
Ler-1 x Rld-1
Ler-1
Ws-0
Ws-0 x Rld-1
Rld-1 x Ws-0
Rld-1
Col
Col x Ler-1
Ler-1 x Col
Ler-1 x Col
Col
Col x Ws-0
Ws-0 x Col
Ws-0
Ws-0/Ler-1
Col/Van-0
Col/Co-1
Col/Sha
Ws-0
Ws-0 x Ler-1
Ler-1 x Ws-0
Ler-1
Col
Col x Van-0
Van-0 x Col
Van-0
Col
Col x Co-1
Co-1 x Col
Co-1
Col
Col x Sha
Sha x Col
Sha
Col/Bch-1
Col/Bay-0
Col/Ak-1
Col/Wl-0
Hours after sowing
Col
Col x Bch-1
Bch-1 x Col
Bch-1
Col
Col x Bay-0
Bay-0 x Col
Bay-0
Col
Col x Ak-1
Ak-1 x Col
Ak-1
Col
Col xWl-0
Wl-0 x Col
Wl-0

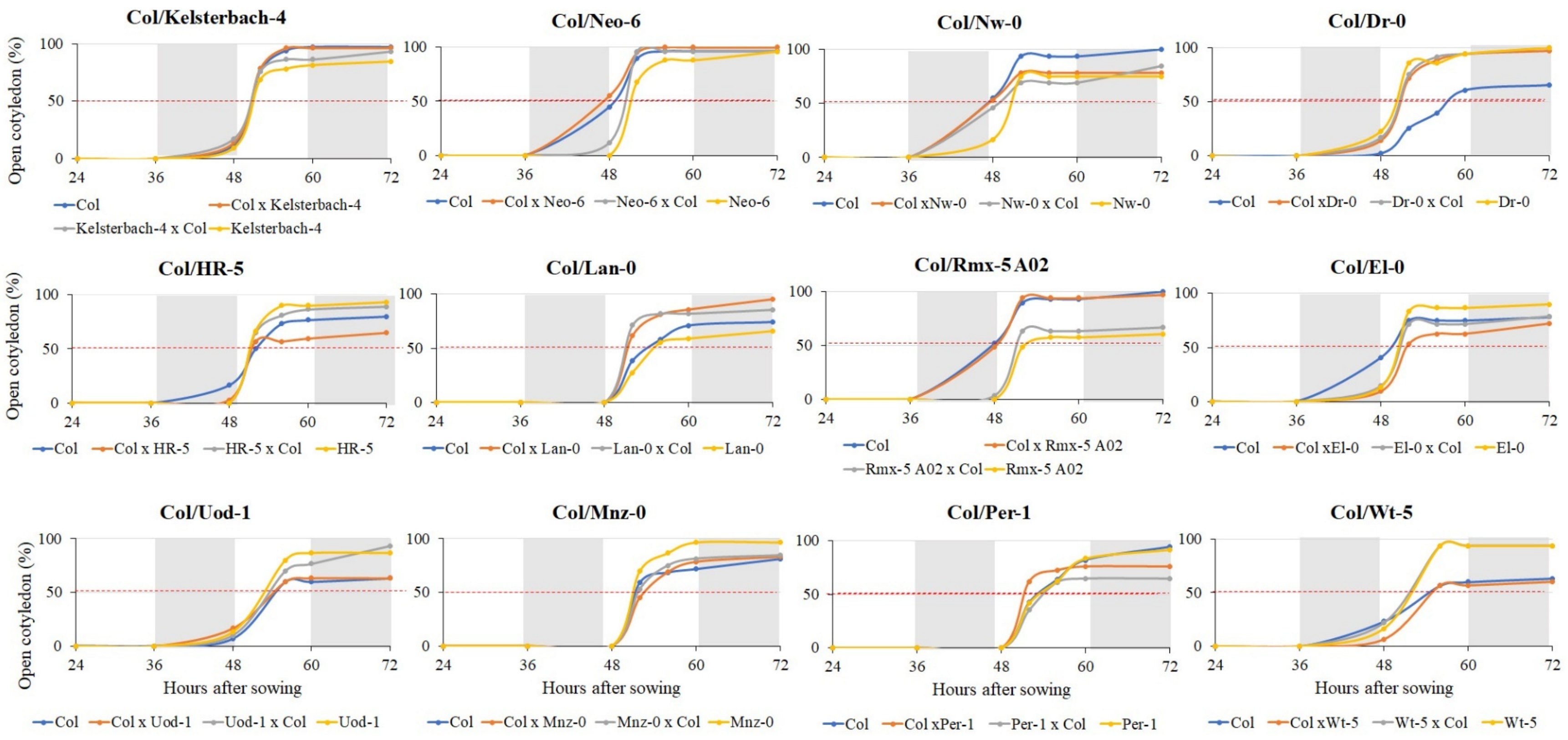

Figure S4. Seed germination time of intraspecific hybrids and their parents.

The percentage of fully open cotyledons was tracked between 24 and 72 HAS. Gray blocks represent the dark phase of the day. Red horizontal lines mean seed germination time (50% fully open cotyledons).

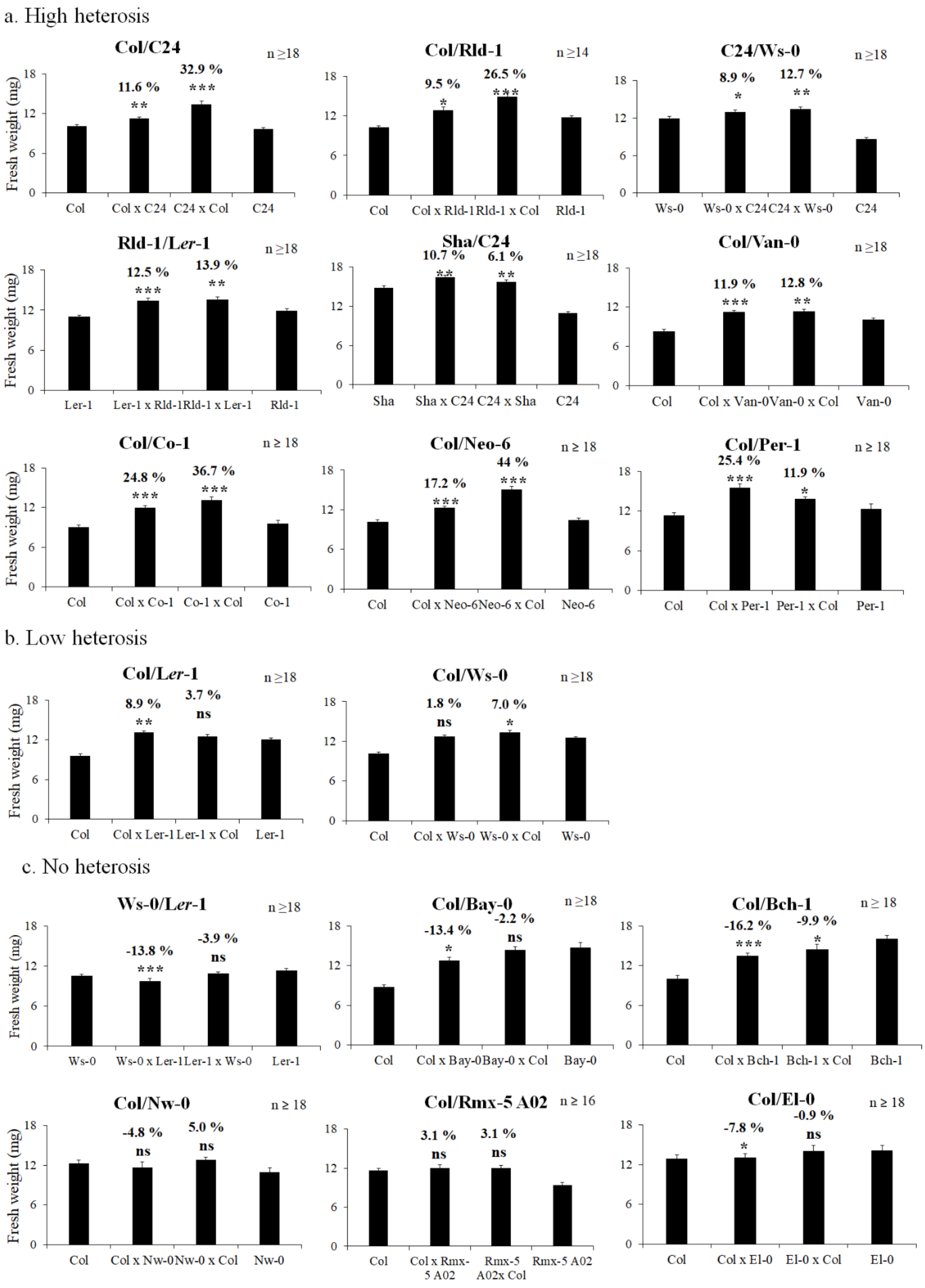
a. High heterosis
Col/C24
n ≥18
11.6 %
**
32.9 %
***
Fresh weight (mg)
Col
Col x C24
C24 x Col
C24
Col/Rld-1
n ≥14
9.5 %
*
26.5 %
***
Col
Col x Rld-1
Rld-1 x Col
Rld-1
C24/Ws-0
n ≥18
8.9 %
*
12.7 %
**
Ws-0
Ws-0 x C24
C24 x Ws-0
C24
Rld-1/Ler-1
n ≥18
12.5 %
***
13.9 %
**
Ler-1
Ler-1 x Rld-1
Rld-1 x Ler-1
Rld-1
Sha/C24
n ≥18
10.7 %
**
6.1 %
**
Sha
Sha x C24
C24 x Sha
C24
Col/Van-0
n ≥18
11.9 %
***
12.8 %
**
Col
Col x Van-0
Van-0 x Col
Van-0
Col/Co-1
n ≥ 18
24.8 %
***
36.7 %
***
Col
Col x Co-1
Co-1 x Col
Co-1
Col/Neo-6
n ≥ 18
17.2 %
***
44 %
***
Col
Col x Neo-6
Neo-6 x Col
Neo-6
Col/Per-1
n ≥ 18
25.4 %
***
11.9 %
*
Col
Col x Per-1
Per-1 x Col
Per-1
b. Low heterosis
Col/Ler-1
n ≥18
8.9 %
**
3.7 %
ns
Col
Col x Ler-1
Ler-1 x Col
Ler-1
Col/Ws-0
n ≥18
1.8 %
ns
7.0 %
*
Col
Col x Ws-0
Ws-0 x Col
Ws-0
c. No heterosis
Ws-0/Ler-1
n ≥18
-13.8 %
***
-3.9 %
ns
Ws-0
Ws-0 x Ler-1
Ler-1 x Ws-0
Ler-1
Col/Bay-0
n ≥18
-13.4 %
*
-2.2 %
ns
Col
Col x Bay-0
Bay-0 x Col
Bay-0
Col/Bch-1
n ≥ 18
-16.2 %
***
-9.9 %
*
Col
Col x Bch-1
Bch-1 x Col
Bch-1
Col/Nw-0
n ≥ 18
-4.8 %
ns
5.0 %
ns
Col
Col x Nw-0
Nw-0 x Col
Nw-0
Col/Rmx-5 A02
n ≥ 16
3.1 %
ns
3.1 %
ns
Col
Col x Rmx-5 A02
Rmx-5 A02x Col
Rmx-5 A02
Col/El-0
n ≥ 18
-7.8 %
*
-0.9 %
ns
Col
Col x El-0
El-0 x Col
El-0

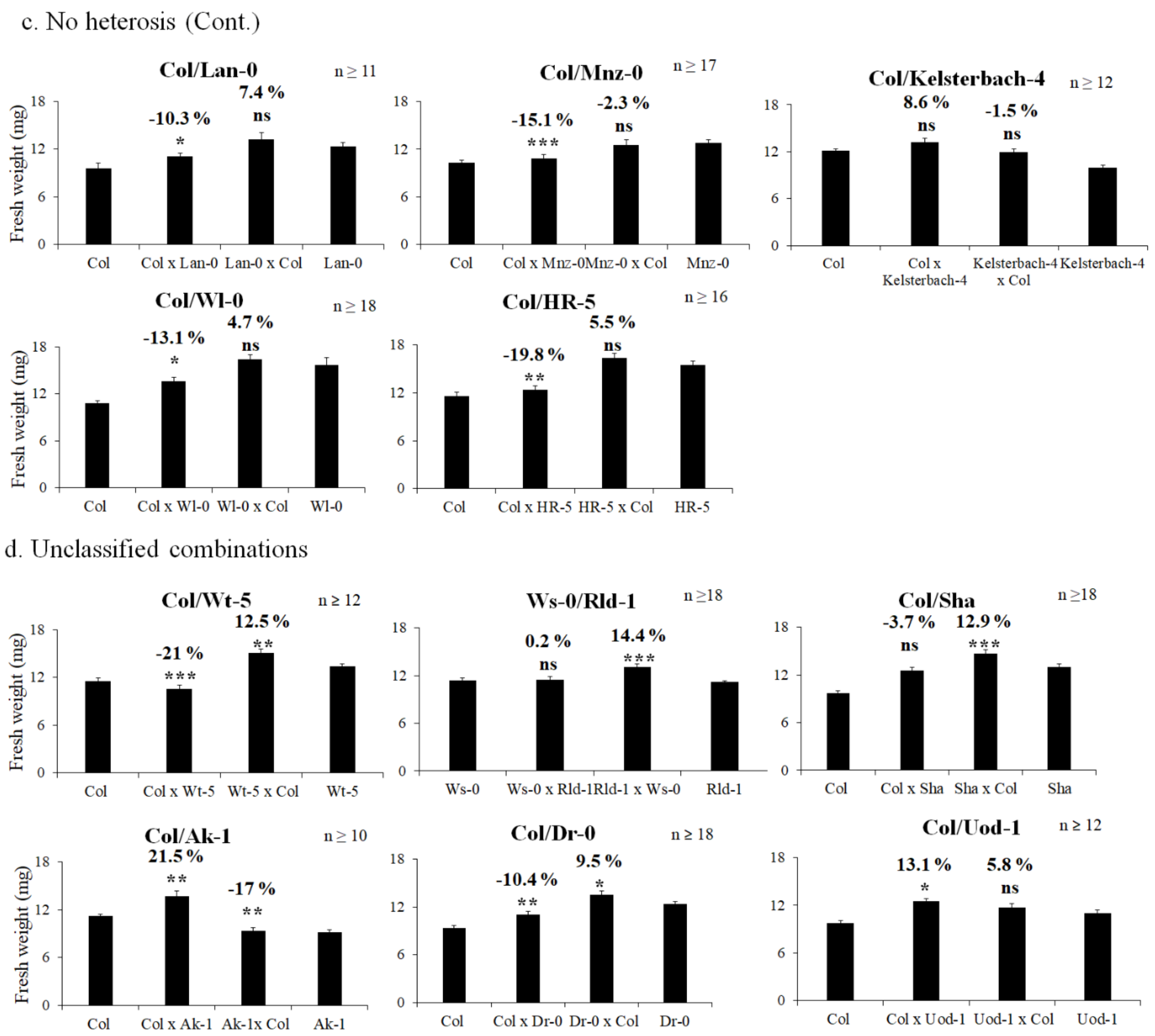

Figure S5. Fresh weight of 28 hybrid combinations at 15 DAS.

High heterosis (a), low heterosis (b), no heterosis (c), and unclassified combinations (d). Numbers above each hybrid's bar represent a level from BPV. Black asterisks show above BPV; ns: not significant, * $P < 0.05$, ** $P < 0.01$, *** $P < 0.001$, Student's *t*-test. n represents in detail the number of plants collected from each combination. Data indicate the average and error bar (standard error).

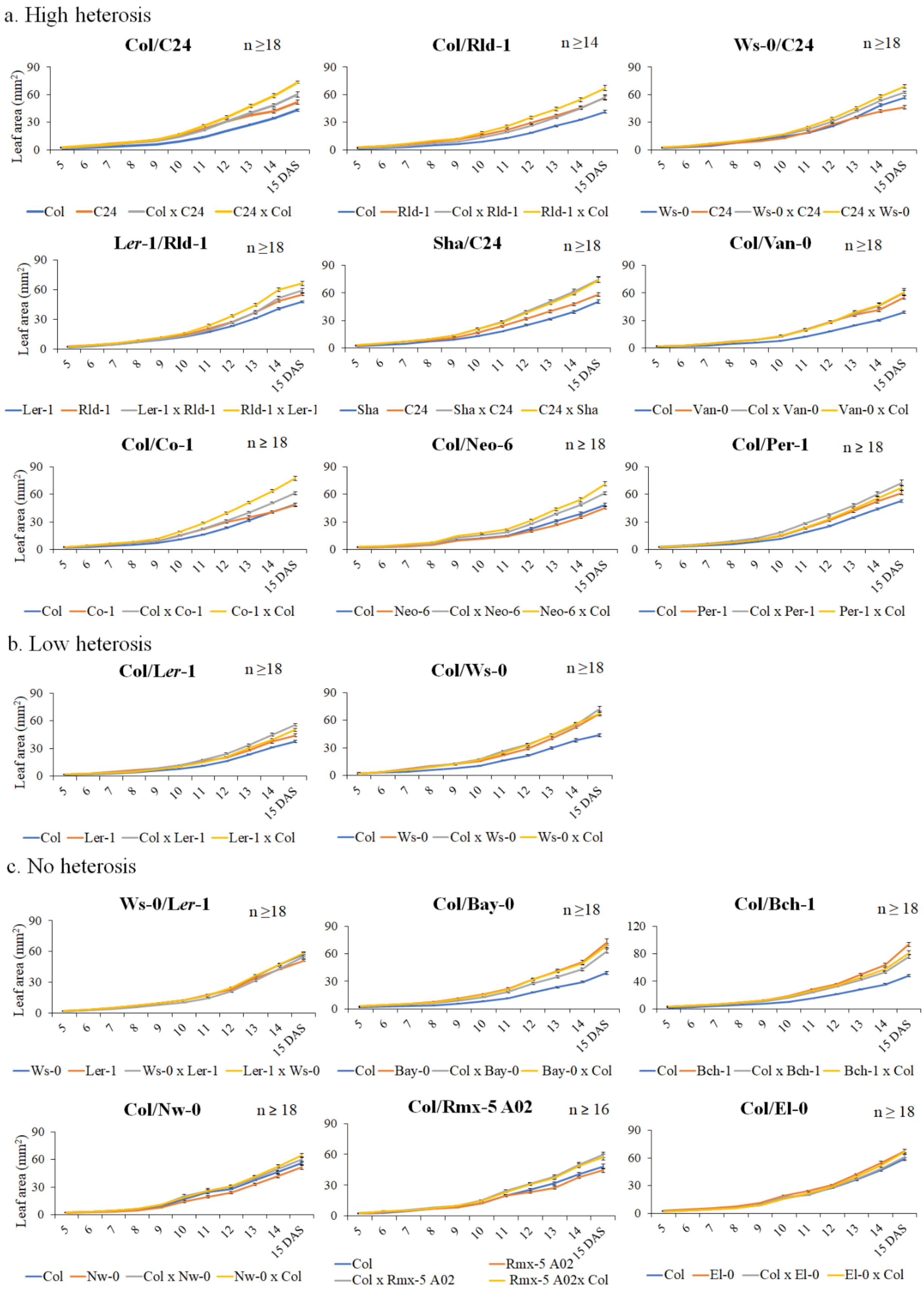

a. High heterosis
Col/C24 n ≥18
Leaf area (mm²)
Col C24 Col x C24 C24 x Col
Col/Rld-1 n ≥14
Col Rld-1 Col x Rld-1 Rld-1 x Col
Ws-0/C24 n ≥18
Ws-0 C24 Ws-0 x C24 C24 x Ws-0
Ler-1/Rld-1 n ≥18
Ler-1 Rld-1 Ler-1 x Rld-1 Rld-1 x Ler-1
Sha/C24 n ≥18
Sha C24 Sha x C24 C24 x Sha
Col/Van-0 n ≥18
Col Van-0 Col x Van-0 Van-0 x Col
Col/Co-1 n ≥ 18
Col Co-1 Col x Co-1 Co-1 x Col
Col/Neo-6 n ≥ 18
Col Neo-6 Col x Neo-6 Neo-6 x Col
Col/Per-1 n ≥ 18
Col Per-1 Col x Per-1 Per-1 x Col
b. Low heterosis
Col/Ler-1 n ≥18
Col Ler-1 Col x Ler-1 Ler-1 x Col
Col/Ws-0 n ≥18
Col Ws-0 Col x Ws-0 Ws-0 x Col
c. No heterosis
Ws-0/Ler-1 n ≥18
Ws-0 Ler-1 Ws-0 x Ler-1 Ler-1 x Ws-0
Col/Bay-0 n ≥18
Col Bay-0 Col x Bay-0 Bay-0 x Col
Col/Bch-1 n ≥ 18
Col Bch-1 Col x Bch-1 Bch-1 x Col
Col/Nw-0 n ≥ 18
Col Nw-0 Col x Nw-0 Nw-0 x Col
Col/Rmx-5 A02 n ≥ 16
Col Rmx-5 A02 Col x Rmx-5 A02 Rmx-5 A02x Col
Col/El-0 n ≥ 18
Col El-0 Col x El-0 El-0 x Col
5 6 7 8 9 10 11 12 13 14 15 DAS

c. No heterosis (Cont.)

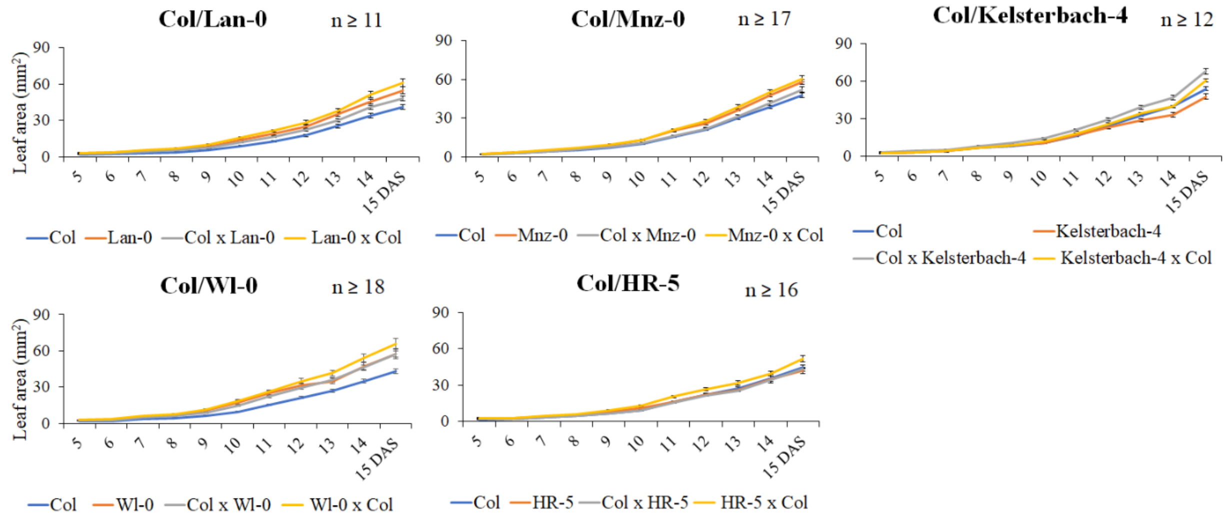

d. Unclassified combinations

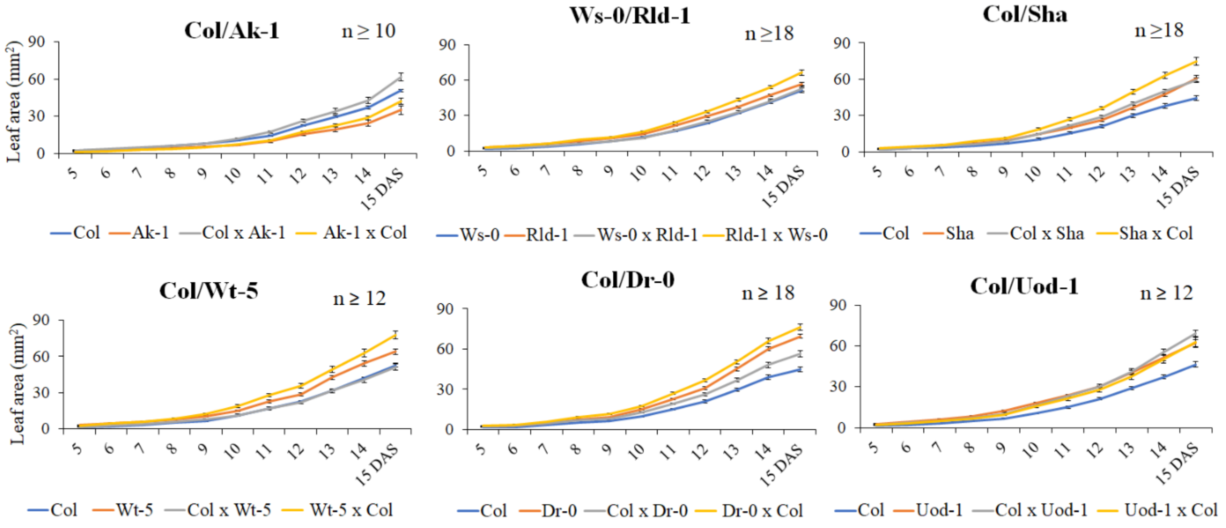

Figure S6. Leaf area of 28 hybrid combinations at 15 DAS. High heterosis (a), low heterosis (b), no heterosis (c), and unclassified combinations (d). n represents the number of plants collected from each combination. Data indicate average and error bar standard error.

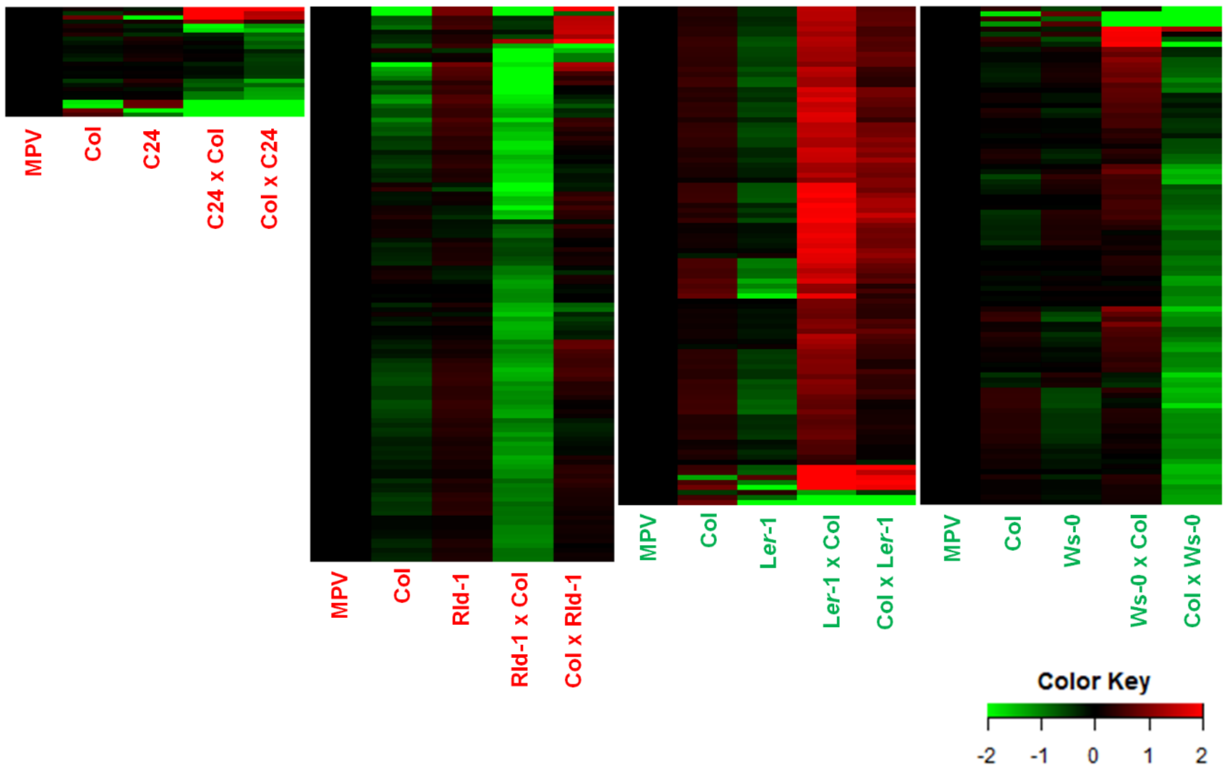

Figure S7. Heat map represents significantly altered metabolites in at least one reciprocal F_1 hybrid from combinations.

Significant comparisons were performed by multiple *t*-tests (FDR < 0.05), increased/decreased metabolites compared with both parents. Differences in red/green colors exhibit the $\log_2$ fold change (up/down) normalized to MPV (control: MPV as 0 in $\log_2$ fold change). Red/green words mean the name of high/low-biomass heterosis.

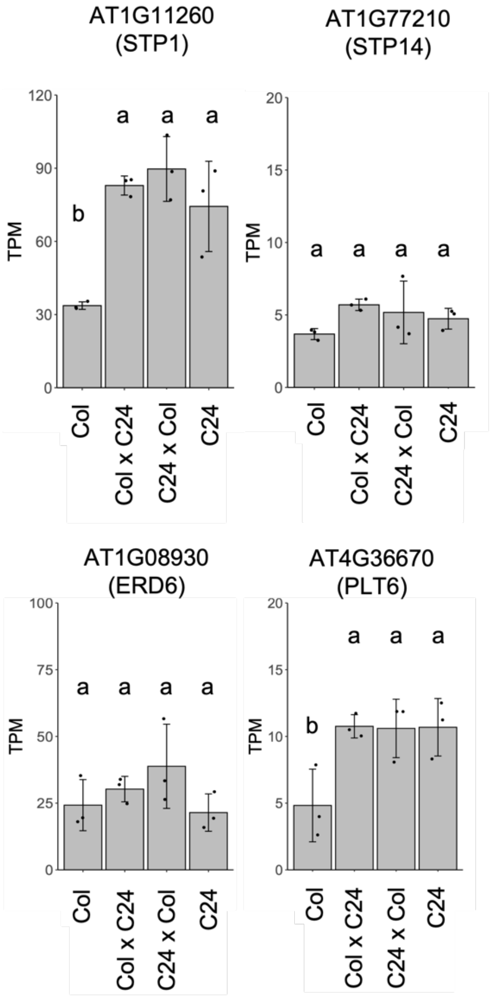

Figure S8. Expression pattern of sugar metabolism-related genes in Col, C24, and their reciprocal F_1 hybrids.

Expression patterns of sugar transporters are mentioned in Groszmann et al. 2015. Different letters indicate significant differences based on Tukey's test (p<0.05). Error bars represent the SD of three biological replicates.

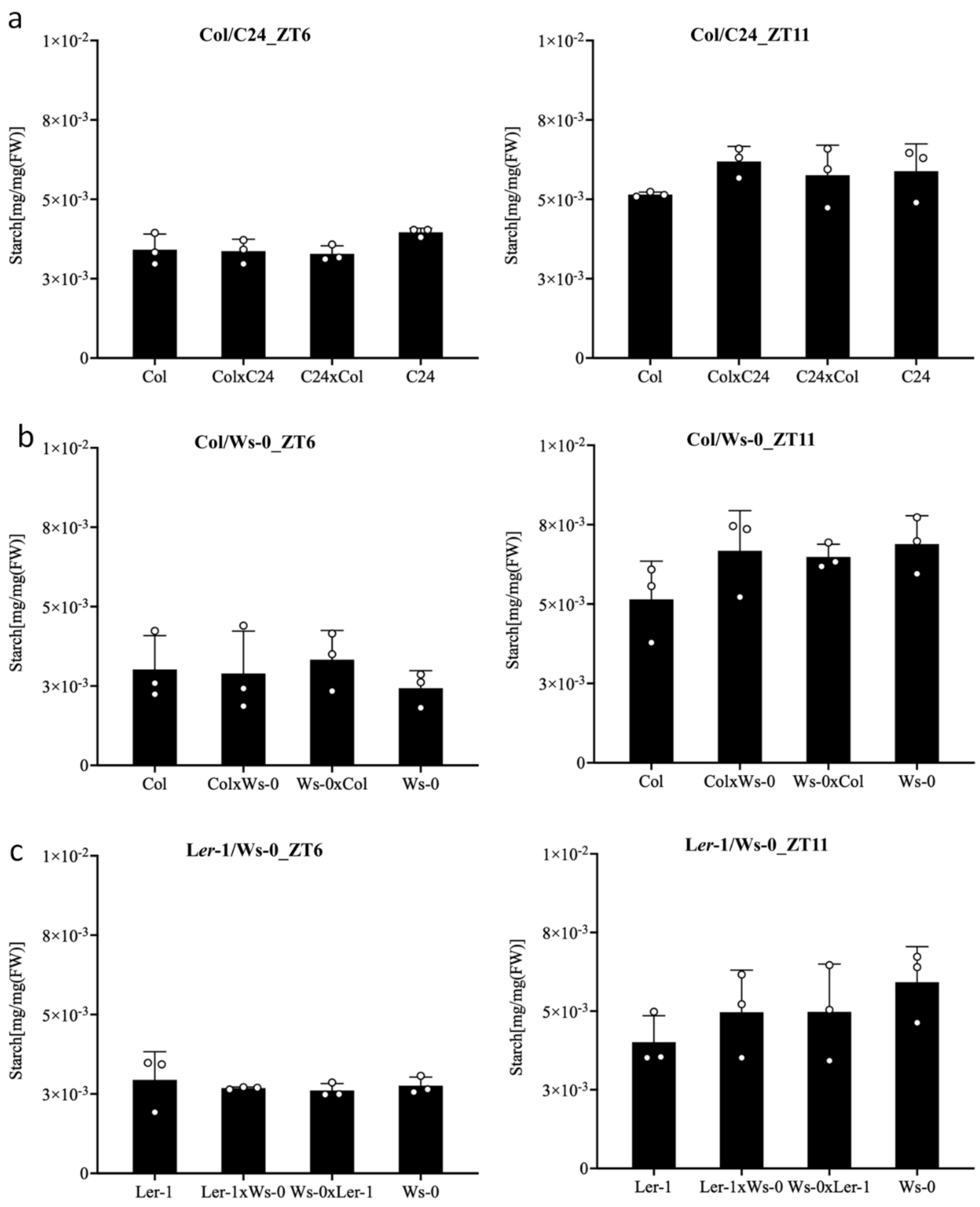

Figure S9. Measurement of starch levels in 15-day-old Arabidopsis seedlings of different heterosis classes.

The hybrids and parents of (a) Col/C24 (high heterosis), (b) Col/Ws-0 (low heterosis), and (c) L*er*-1/Ws-0 (no heterosis). Rosettes of 15 DAS seedlings were harvested at ZT6 and ZT11, respectively. The data shown were the average and SD (n = 3).

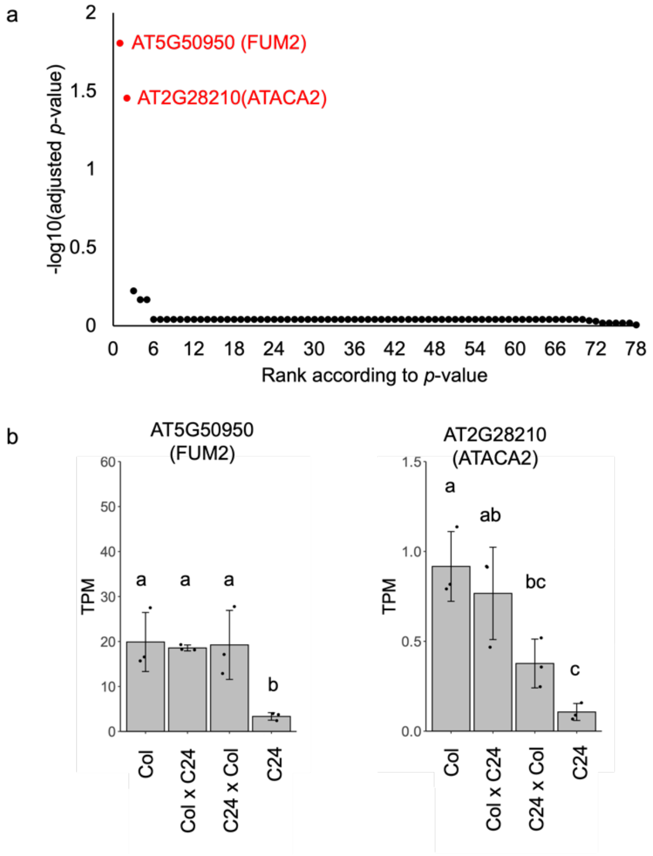

Figure S10. Expression patterns of genes related to TCA cycle in Col, C24 and their reciprocal F_1 hybrids.

a: Overview of statistical analysis in TCA cycle-related genes. One-way ANOVA was performed among Col, C24, and their F_1 hybrids to calculate a raw *p*-value. FDR-adjusted *p*-values were calculated by R software.

b: Differentially expressed genes related to the TCA cycle. Different letters indicate significant differences based on Tukey's test ($p<0.05$). Error bars represent the SD of three biological replicates.

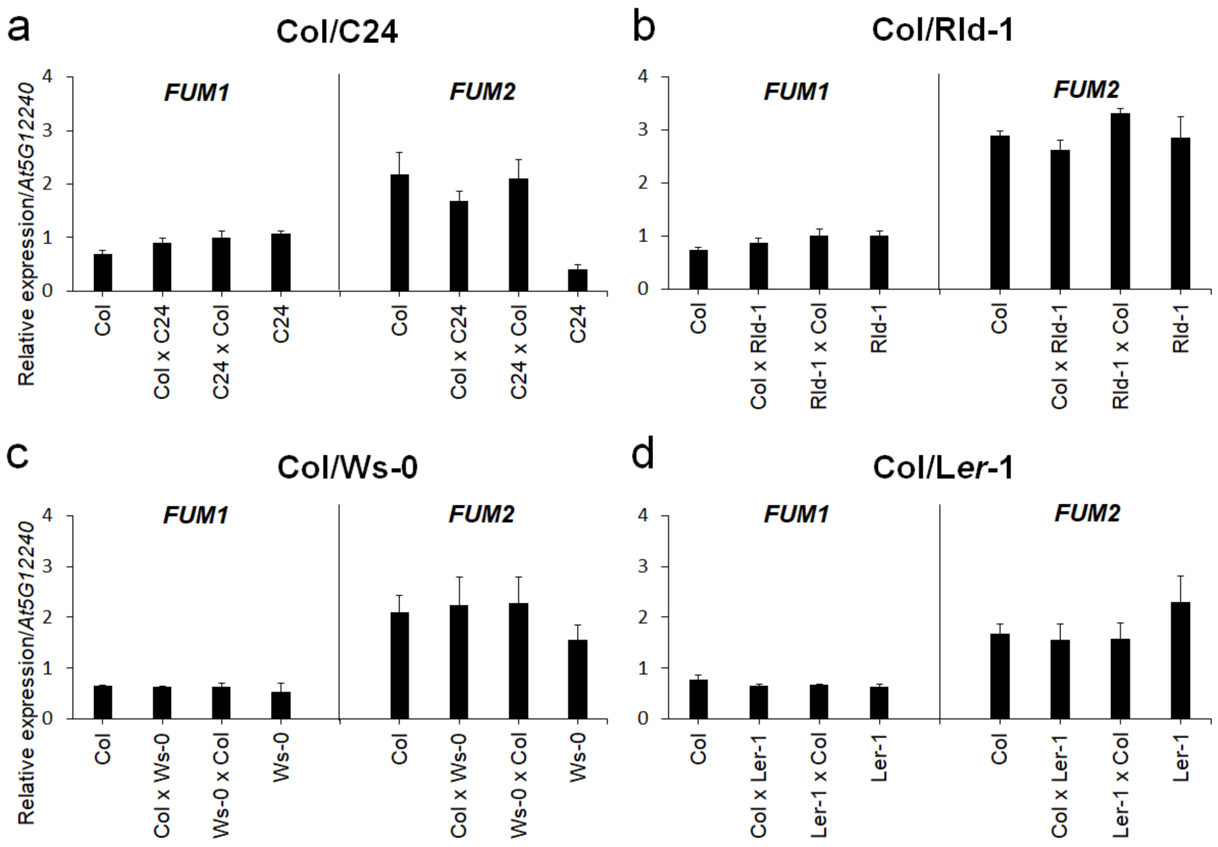

Figure S11. Gene expression of *FUM1* and *FUM2* in the different combinations.

The hybrids and parents of (a) Col/C24, (b) Col/Rld-1, (c) Col/Ws-0, and (d) Col/L*er*-1 combination.

Data represent the average of three biological replicates and standard error.
